# Identifying collagen VI as a target of fibrotic diseases regulated by CREBBP/EP300

**DOI:** 10.1073/pnas.2004281117

**Published:** 2020-08-05

**Authors:** Lynn M. Williams, Fiona E. McCann, Marisa A. Cabrita, Thomas Layton, Adam Cribbs, Bogdan Knezevic, Hai Fang, Julian Knight, Mingjun Zhang, Roman Fischer, Sarah Bonham, Leenart M. Steenbeek, Nan Yang, Manu Sood, Chris Bainbridge, David Warwick, Lorraine Harry, Dominique Davidson, Weilin Xie, Michael Sundstrӧm, Marc Feldmann, Jagdeep Nanchahal

**Affiliations:** ^a^Kennedy Institute of Rheumatology, Nuffield Department of Orthopaedics, Rheumatology and Musculoskeletal Science, University of Oxford, Oxford OX3 7FY, United Kingdom;; ^b^Botnar Research Centre, National Institute for Health Research Oxford Biomedical Research Unit, Nuffield Department of Orthopaedics, Rheumatology and Musculoskeletal Science, University of Oxford, Oxford OX3 7LD, United Kingdom;; ^c^Wellcome Trust Centre for Human Genetics, University of Oxford, Oxford OX3 7BN, United Kingdom;; ^d^Biotherapeutics Department, Celgene Corporation, San Diego, CA 92121;; ^e^Target Discovery Institute, Nuffield Department of Medicine, University of Oxford, Oxford OX3 7FZ, United Kingdom;; ^f^Department of Plastic Surgery, Geert Grooteplein Zuid 10, 6525 GA Nijmegen, The Netherlands;; ^g^Department of Plastic and Reconstructive Surgery, Broomfield Hospital, Mid and South Essex National Health Service Foundation Trust, Chelmsford CM1 4ET, Essex, United Kingdom;; ^h^Pulvertaft Hand Surgery Centre, Royal Derby Hospital, University Hospitals of Derby and Burton National Health Service Foundation Trust, Derby DE22 3NE, United Kingdom;; ^i^Department of Trauma and Orthopaedic Surgery, University Hospital Southampton National Health Service Foundation Trust, Southampton SO16 6YD, United Kingdom;; ^j^Department of Plastic and Reconstructive Surgery, Queen Victoria Hospital National Health Service Foundation Trust, East Grinstead RH19 3DZ, United Kingdom;; ^k^Department of Plastic and Reconstructive Surgery, St. John’s Hospital, Livingston, West Lothian EH54 6PP, United Kingdom;; ^l^Structural Genomics Consortium, Karolinska Centre for Molecular Medicine, Karolinska University Hospital, 171 76 Stockholm, Sweden

**Keywords:** EP300, fibrosis, collagen VI, epigenetic

## Abstract

Fibrotic diseases remain a major cause of morbidity and mortality, yet there are few effective therapies. The underlying pathology of all fibrotic conditions is the activity of myofibroblasts. Using cells from freshly excised disease tissue from patients with Dupuytren’s disease (DD), a localized fibrotic disorder of the palm, we sought to identify new therapeutic targets for fibrotic disease. We hypothesized that the persistent activity of myofibroblasts in fibrotic diseases might involve epigenetic modifications. Using a validated genetics-led target prioritization algorithm (Pi) of genome wide association studies (GWAS) data and a broad screen of epigenetic inhibitors, we found that the acetyltransferase CREBBP/EP300 is a major regulator of contractility and extracellular matrix production via control of H3K27 acetylation at the profibrotic genes, *ACTA2* and *COL1A1*. Genomic analysis revealed that EP300 is highly enriched at enhancers associated with genes involved in multiple profibrotic pathways, and broad transcriptomic and proteomic profiling of CREBBP/EP300 inhibition by the chemical probe SGC-CBP30 identified collagen VI (Col VI) as a prominent downstream regulator of myofibroblast activity. Targeted Col VI knockdown results in significant decrease in profibrotic functions, including myofibroblast contractile force, extracellular matrix (ECM) production, chemotaxis, and wound healing. Further evidence for Col VI as a major determinant of fibrosis is its abundant expression within Dupuytren’s nodules and also in the fibrotic foci of idiopathic pulmonary fibrosis (IPF). Thus, Col VI may represent a tractable therapeutic target across a range of fibrotic disorders.

## Significance

Fibrosis remains a major unmet medical need as therapeutic targets discovered in animal models have failed to translate. A major challenge for identifying novel targets is the limited availability of early-stage human disease tissue. Here, we utilize Dupuytren’s disease (DD), a common localized fibrotic disorder, to evaluate the impact of epigenetic regulation of myofibroblasts and identify potential tractable targets in human fibrosis. We demonstrate that the epigenetic regulator CREBBP/EP300 is a critical determinant of the profibrotic phenotype. Furthermore, we identify collagen VI to be a key downstream target of CREBBP/EP300 and reveal valuable insights in the role it plays in key profibrotic functions, including contractile force, chemotaxis, and wound healing, and hence its potential as a therapeutic target.

Fibrosis of visceral organs such as the lungs, heart, kidneys, and liver is a major cause of morbidity and mortality (1). However, despite intense research efforts, little progress has been made in the identification of effective therapeutic targets. Potential reasons for this include late presentation of patients with fibrotic diseases involving internal organs, lack of good disease biomarkers, and poor translation through to clinical practice of targets identified in murine models (2). A greater understanding of the mechanisms controlling the fibrotic pathways in human tissues is required to facilitate the development of next-generation therapies.

Dupuytren’s disease (DD) is a debilitating fibroproliferative disorder restricted to the palms of genetically susceptible individuals (3). The initial clinical presentation is the appearance of a firm cellular nodule in the hand, which expands into fibrous collagenous cords that extend into the digits. As the disease progresses, the relatively acellular cords mature, thicken, and contract, leading to permanent flexion deformities of the fingers. Unlike fibrotic diseases of visceral organs such as the lung and liver which are diagnosed late, DD tissue can be diagnosed early and is readily accessible for research as the disease is often treated by surgical excision. Using the relatively early-stage nodules from patients with Dupuytren’s disease enables us to analyze signaling and regulatory pathways and hence has the potential for the identification of novel therapeutic targets. Dupuytren’s nodules represent a complex disease system, housing densely packed myofibroblasts (MFs) alongside other less abundant stromal and immune cells (4). Myofibroblasts are characterized by the expression of α-smooth muscle actin (α-SMA) and excessive production of extracellular matrix (ECM) proteins such as collagens, glycoproteins, and proteoglycans, which enhances their ability to contract tissue (5, 6). Matrix stiffness and mechanical stress in severely damaged tissues initiates a positive feedback loop between myofibroblasts and their surrounding microenvironment that perpetuates fibrosis (7, 8). Therefore, Dupuytren’s nodules provide an ideal model system to study primary human myofibroblasts, the cell type responsible for deposition and contraction of excessive matrix in all forms of fibrosis (9, 10).

Despite the substantial heritability of 80% (11) in DD and multiple risk loci identified by genome wide association studies (GWAS) (12), why this disease is restricted to the palmar fascia of the hand is still poorly understood. The distinct anatomical location and proposed role of environmental factors such as excessive alcohol consumption (13), heavy manual labor, and exposure to vibrations (14) suggest epigenetic regulation is likely to be important in the pathogenesis of the disease. Epigenetic mechanisms have been shown to be crucial mediators of fibrotic gene expression in a wide range of fibrotic disorders (15), including cardiac fibrosis (16), liver fibrosis (17), and systemic sclerosis (18), and probably account for the persistence of myofibroblasts in disease (19).

We hypothesized that specific targeting of transcriptional coactivators could provide valuable insight into epigenetic mechanisms controlling the myofibroblast phenotype in fibrosis. Here we show that the histone acetyltransferases CREBBP and EP300 (20) are key regulators of profibrotic phenotype and function of myofibroblasts and act through control of collagen VI. Furthermore, collagen VIα3 is highly expressed in both Dupuytren’s nodules and in the fibroblastic foci in idiopathic pulmonary fibrosis (IPF), with a distribution distinct from collagen I but closely aligned with α-SMA positive regions, thus emerging as a signature marker of myofibroblasts and a tractable therapeutic target for DD and potentially also other fibrotic diseases.

## Results

### Computational Target Prioritization and Screening Using Pharmacological Inhibitors Both Identify *CREBBP*/*EP300* as a Central Regulatory Network in Human Fibrosis.

**Fig. 1.**
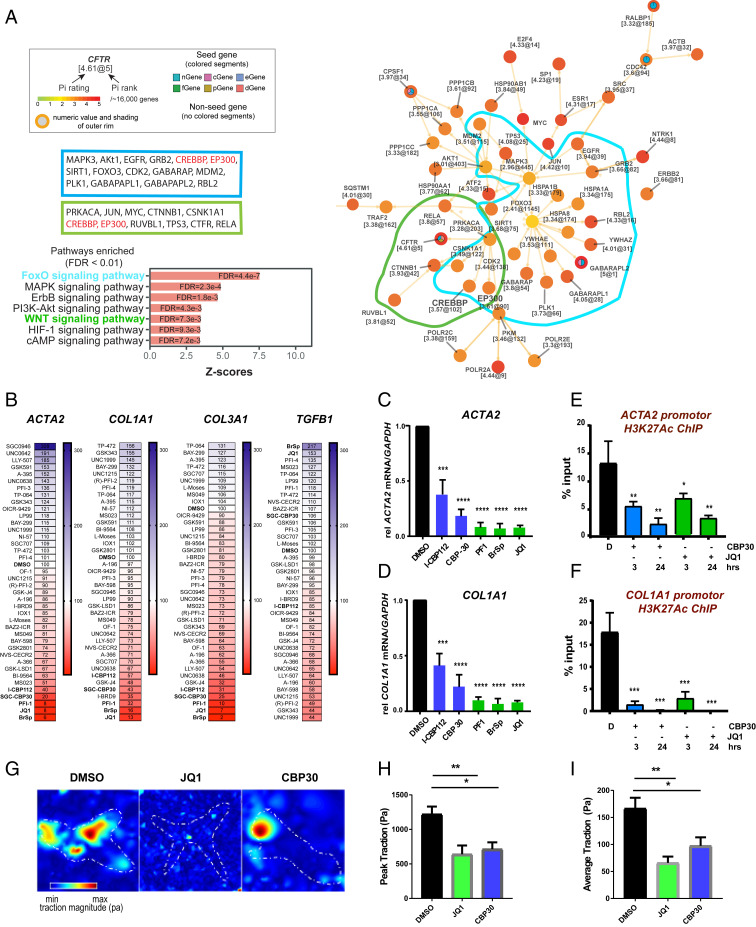
CREBBP/EP300 regulates profibrotic gene expression and contractile phenotype in myofibroblasts. (*A*) Pi analysis identifies CREBBP and EP300 as highly ranked central network genes in DD. Target pathway crosstalk for DD is shown, maximizing numbers of highly prioritized interconnecting genes. (*B*) Epigenetic probe library screening of *ACTA2*, *COL1A1*, *COL3A1*, and *TGFB1* gene expression in DD patient-derived myofibroblasts. Cells were incubated with probes for 3 d. Gene expression was analyzed by Taqman qPCR using the ∆∆Ct method, normalized to *GAPDH*. Data are means ± SEM of five independent experiments from five individual donors, represented as a heatmap where values are normalized to DMSO control value; 100% represented in white, red represents inhibition and blue represents augmentation of gene expression relative to control. (*C* and *D*) Data from heatmap where the most potent inhibitory probes are depicted graphically; mean ± SEM from five donors. *P* value was determined by one-sample *t* test to normalized control (DMSO) sample of value of 1. *P ≤ 0.05, **P < 0.01, ***P < 0.001, ****P < 0.0001. ChIP-qPCR analysis of JQ1 (0.5 µM) and SGC-CBP30 (2.5 µM) inhibition of the H3K27ac mark at promotor proximal regions of *ACTA2* (*E*) and *COL1A1* (*F*) in patient-derived myofibroblasts. Data are representative of three independent experiments from three individual donors, mean ± SD of technical triplicates. Myofibroblasts were plated onto 2.55-kPa hydrogels for traction force microscopy in the presence of the indicated bromodomain inhibitors for 72 h. Color scale in stiffness maps indicates shear modulus in kilopascals. (*G*) Representative traction vector maps, (*H*) peak traction, and (*I*) average traction are depicted (mean ± SEM; *n* = 4 independent experiments with a minimum of 20 independent cells per condition; **P < 0.01 and *P < 0.05 versus controls).

To leverage the power of genetic variance in target discovery, we applied our recently developed prioritization “Pi” approach (21) to risk loci in DD GWAS data (12). This validated approach allows us to prioritize genes from GWAS hits, not simply based on genomic proximity but also including evidence from chromatin conformation and expression quantative trait loci mapping in immune cells, plus gene networks defined by the Search Tool for the Retrieval of Interacting Genes/Proteins database. This unsupervised network connectivity analysis identified FoxO and WNT signaling as highly ranked pathways that share *CREBBP* and *EP300* member genes, as shown at the intersection of delineated pathways in blue and green, respectively (Fig. 1*A* and *SI Appendix*, Table S1), providing unbiased support for our hypothesis that epigenetic modulators are important in controlling disease processes in DD.

For functional validation, we screened a focused library of 39 epigenetic inhibitors (*SI Appendix*, Table S2) to study their effect on the expression of key profibrotic genes (*ACTA2*, *COL1A1*, *COL3A1*, and *TGFB1*) on early passage myofibroblasts derived from nodules in patients with DD (Fig. 1*B* and *SI Appendix*, Fig. S1). This revealed the pan bromodomain inhibitor bromosporine (BrSp), the BET bromodomain inhibitors, PFI-1 and JQ1, and the two structurally distinct CREBBP/EP300 bromodomains inhibitors SGC-CBP30 (referred to as CBP30 throughout) and I-CBP112, as the most potent and significant inhibitors of *ACTA2* and *COL1A1* gene expression (Fig. 1*B*), with a strong dose-dependent effect (*SI Appendix*, Fig. S1). We also employed a recently developed, highly specific CBP/EP300 HAT inhibitor A485 (22), to confirm the role of CBP/EP300-mediated histone acetylation regulating *ACTA2* and *COL1A1* gene expression (*SI Appendix*, Fig. S1 *G*–*I*).

To establish whether pharmacologic inhibition of CBP/EP300 bromodomains would impact histone acetylation at the transcription starts sites of *ACTA2* and *COL1A1*, we employed chromatin immunoprecipitation (ChIP) qPCR with antibodies for the H3K27ac mark. We also included the BET inhibitor JQ1 as it also reduced expression of *ACTA2* and *COL1A1* in our primary qPCR screen. Both CBP30 and JQ1 treatment reduced H3K27 acetylation at the transcriptional start sites of *ACTA2* and *COL1A1*, confirming a direct role in regulating gene transcription (Fig. 1 *E* and *F*).

Since excessive myofibroblast contractility is a key feature in disease pathogenicity in vivo, we measured the effects of JQ1 and CBP30 on myofibroblast force generation as a function of contractility using traction force microscopy (23). This demonstrated that both compounds significantly inhibited cell contractility, with both peak and average traction force reduced by ∼50% (Fig. 1 *G*–*I*). In addition, both inhibitors reduced cell proliferation (*SI Appendix*, Fig. S1*A*), with JQ1, but not CBP30, also reducing cell viability and confluency (*SI Appendix*, Fig. S1 *B* and *C*). Myofibroblasts display spindle or stellate-cell morphology, and while treatment with CBP30 had no clear effect on myofibroblast morphology, JQ1 treatment also altered cell shape, resulting in an elongated neuron-like morphology, indicative of broad effects not shared with CBP30 (*SI Appendix*, Fig. S2 *D*–*G*). Collectively, these data indicate CREBBP/EP300 is a highly associated pathway in localized human fibrosis that controls key aspects of myofibroblast phenotype and function, including *ACTA*2 and *COL1A1* gene expression and cell contractility.

### *EP300* Associates with Active Enhancers in Human Myofibroblasts.

**Fig. 2.**
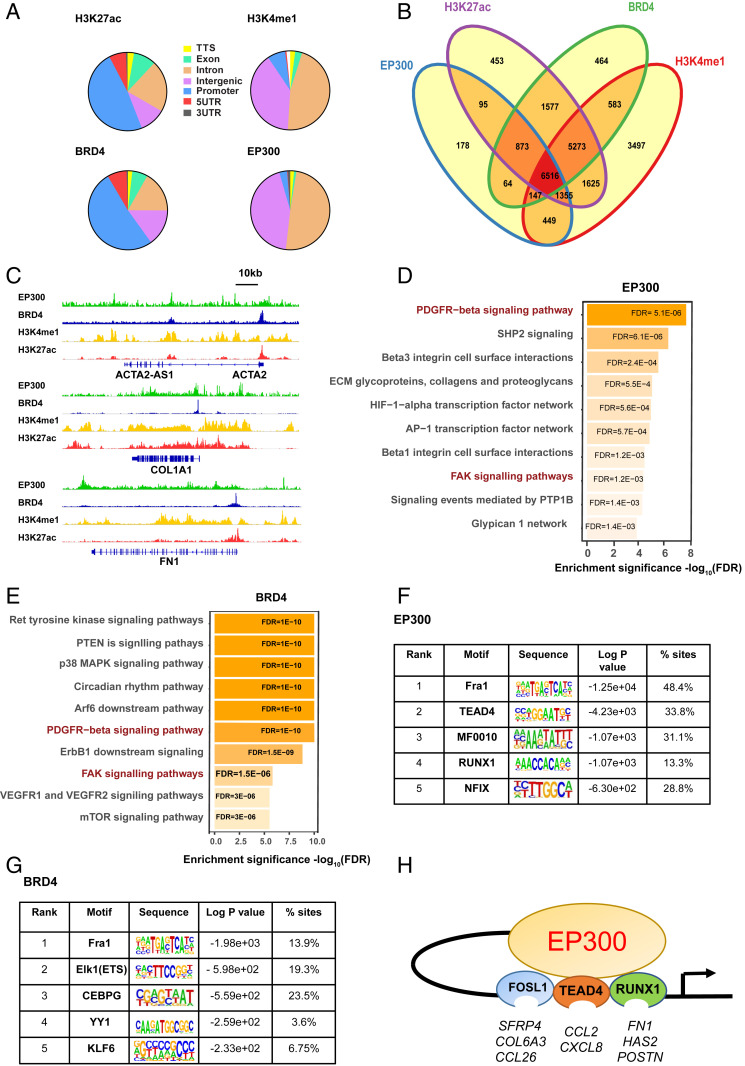
EP300 associates with active enhancers. (*A*) Genomic distribution of H3K27ac, H3K4me1, BRD4, and EP300 peaks. (*B*) Venn diagram of overlap of H3K27ac, H3K4me1, BRD4, and EP300 peaks as determined by Homer AnnotatePeaks. (*C*) Visualization in IGV of ChIP-Seq signal at the *ACTA2*, *COL1A1*, and *FN1* loci; for scaling, group autoscaling was used; all files were normalized to input. (*D*) XGR gene enrichment analysis of canonical pathway analysis of EP300 and (*E*) BRD4 peaks. De novo motif analysis of (*F*) EP300 and (*G*) BRD4-bound genomic regions showing top enriched sequence motifs. *P* values and frequencies are indicated. (*H*) Schematic representing transcription factor-dependent gene expression. FDR, false discovery rate.

Transcriptional regulation of gene expression is achieved through cooperation between promoter and enhancer elements. Specific histone modifications function as binding elements for effector proteins that serve to regulate transcription through manipulation of the chromatin environment or assembly of transcription machinery. Acetylation at lysine 27 of histone H3 serves as a signature mark of active enhancers (24) in all cell types, where monomethylation of histone H3 at lysine 4 (H3K4me1) marks both primed and active enhancers in a cell-type-specific manner (25, 26). To further delineate the molecular mechanisms underlying the functional impact of the bromodomain inhibitors CBP30 and JQ1, we performed ChIP analysis coupled with deep sequencing using H3K27ac-, H3K4me1-, EP300-, and BRD4-specific antibodies (27). Genomic distribution of H3K27ac and BRD4 were strikingly similar (Fig. 2*A*) in many genomic regions, in accordance with previous data (28). In contrast, H3K4me1 and EP300 displayed similar genomic distribution, with genomic occupancy assigned predominantly to intronic and intergenic regions. However, there was also significant overlap of genomic occupancy between the datasets; EP300 shared 91% of sites with H3K27ac, 87% with H3K4me1, and 78% with BRD4 (Fig. 2*B*).

We next probed the ChIP sequencing (ChIP-Seq) datasets to verify EP300 binding within the genomic loci of all of the genes identified within the FoxO (blue) and WNT (green) pathways, previously identified from the Pi pathway analysis in Fig. 1*A*. All genomic loci, with the exception of *RELA* and *AKT1* identified EP300 binding within 50 kB of the annotated genes. We have shown two genes each, *FOXO3* and *EGFR* from the FoxO pathway and *JUNB* and *CSNK1A1* from the WNT pathway in *SI Appendix*, Fig. S3, as an illustration of these findings.

We next sought to establish the direct binding of EP300 and BRD4 to key profibrotic genes. We found strong evidence of active transcription at the loci of *ACTA2*, *COL1A1*, and *FN1* (Fig. 2*C*), with H3K27ac and BRD4 highly enriched at the transcriptional start site of all three genes. In contrast, EP300 and H3K4me1 exhibited binding at more distal locations, indicative of enhancer activity. Taken together these data confirm the role of EP300 as an enhancer regulating the expression of *ACTA2* and *COL1A1*.

Several groups have shown that much of disease-associated DNA sequence variation occurs in transcriptional regulatory regions defined by DNase hypersensitivity (29, 30). It has now been demonstrated using multiple GWAS datasets that a subset of (super) enhancers are especially important for genes associated with cell identity and genetic risk of disease (31, 32). Given that EP300 loading identifies regions of the genome bearing super enhancer architecture in all cell types, we sought to determine whether EP300 binding was associated with the single polynucleotide polymorphisms (SNPs) we have previously identified in Dupuytren’s disease GWAS datasets. We analyzed the genomic location of EP300 at the 22 SNPs significantly associated with Dupuytren’s disease (12). As shown in *SI Appendix*, Fig. S4 and summarized in *SI Appendix*, Table S2, EP300 is very closely associated with genomic regions of 8 of the 22 regions, independent of H3K27ac or H3K4me1 binding.

Pathway analysis of annotated ChIP-Seq peaks demonstrated that the EP300 binding (Fig. 2*D*) sites are most significantly enriched for PDGFRβ, integrin, and ECM interaction pathways. In contrast, BRD4 peaks displayed enrichment for a myriad of diverse signaling pathways. Notably, we observed few common pathways enriched across annotated BRD4 and EP300 peaks, suggesting a distinction of biological themes between the two epigenetic regulators, with the exception of PDGFRβ and FAK signaling pathways (Fig. 2*E*; Dataset S1), both of which are highly associated with fibrosis in multiple organs (33). Next, we performed de novo motif analysis of the EP300-enriched loci (Fig. 2*F*). Ranking the motifs by enrichment, the top five corresponded to the known consensus binding sequences for the transcription factors FRA1, TEAD4, MF0010, RUNX1, and NFIX. Finally, motif analysis of the BRD4 dataset also identified FRA1 as the highest number of sites, with ELK1 (ETS), CEBPG, YY1, and KLF6 also significantly enriched (Fig. 2*G*).

To investigate a putative role for the EP300-regulated transcription factors in profibrotic gene expression, we performed siRNA-mediated gene knockdown of Fra1/*FOSL1*, *RUNX1*, and *TEAD4* in DD myofibroblasts and measured the expression of a panel of profibrotic genes (*SI Appendix*, Table S3). This demonstrated that knockdown of *FOSL1* significantly increased expression of several genes, including *CXCL8*, *IL6*, and *CTGF* (*SI Appendix*, Fig. S6 *A*–*C*) and inhibited *CCL26*, *SFRP4*, and *COL6A3* expression (*SI Appendix*, Fig. S5 *D*–*G*). In contrast, *TEAD4* knockdown inhibited mRNA expression of *CXCL8* and *CCL2* (*SI Appendix*, Fig. S5 *H*–*J*), while *RUNX1* knockdown significantly down-regulated mRNA expression of genes involved in ECM remodeling, including fibronectin-1 (*FN1*) and hyaluronan synthase 2 (*HAS2*) (*SI Appendix*, Fig. S5 *K*–*M*). Together, these data support the role of EP300 in modulating diverse profibrotic pathways and identified RUNX1, TEAD4, and Fra1 as EP300-associated transcription factors regulating discrete gene modules in myofibroblasts as summarized in Fig. 2*H*.

### Inhibition with CBP30 Identifies Collagen VI as a Downstream Therapeutic Target of Multiple CBP30-Regulated Core ECM Genes in Fibrosis.

**Fig. 3.**
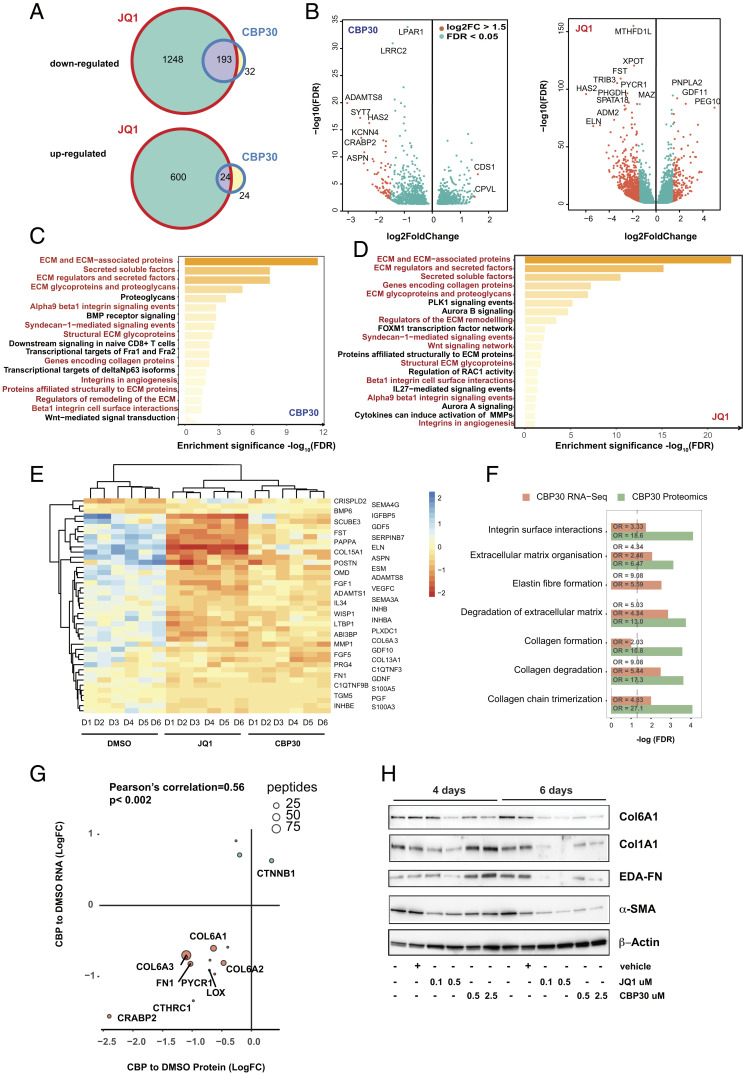
CBP30 treatment targets a limited subset of genes enriched in ECM-mediated regulated pathways. (*A*) Venn diagram showing the number of genes with significantly different expression (log2 FC > 1, FDR < 0.05) following JQ1 (0.5 µM) and CBP30 (2.5 µM) treatment. (*B*) Volcano plots of differentially expressed genes; data are generated for six donors. (*C*) Gene enrichment analysis of canonical pathways of significantly differentially expressed genes treated with CBP30 or (*D*) JQ1 treatment for 3 d. (*E*) Heatmap of genes identified within the ECM and ECM-associated proteins following JQ1 and CBP30 treatment from six donors. (*F*) Pathway analysis of RNA-Seq and proteomic analysis datasets following 3 d of drug treatment; data are mean of six donors with significantly different expression (log2 FC > 1.5, FDR < 0.05). (*G*) Correlation between RNA-Seq and proteomic datasets. (*H*) Myofibroblasts were treated with either JQ1 (0.1 to 0.5 µM) or CBP30 (0.5 to 2.5 µM) for either 4 or 6 d; protein levels of proteomic hits collagen I α1, EDA-fibronectin, and collagen VI α1 and α-SMA were determined via Western blotting by SDS/PAGE on 4 to 20% Tris-glycine polyacrylamide gradient; data are representative of three donors.

After establishing that EP300 interacts directly with enhancer sites on key profibrotic genes, we evaluated changes in the transcriptomic profile following CBP/EP300 inhibition using CBP30 in human DD myofibroblasts and compared with JQ1 treatment as a bromodomain inhibitor (Fig. 3 *A* and *B*) (34). JQ1 resulted in extensive transcriptional changes, 1,441 down-regulated, 624 up-regulated (Dataset S2). In contrast, bromodomain inhibition with CBP30 affected a much smaller set of genes (225 down-regulated versus 48 up-regulated genes, Fig. 3 *A* and *B*). Gene enrichment (canonical) pathway analysis (Dataset S3) indicated that CBP30 down-regulated genes were significantly enriched in ECM and proteoglycan-associated protein pathways, integrin signaling, and syndecan-1 pathways (Fig. 3*C* and Dataset S3), in common with JQ1. Pathways unique to JQ1 included the Aurora A/B, PLK1, and FOXM1 pathways (Fig. 3*D*), consistent with the potent antiproliferative properties of JQ1 (35, 36).

Focusing on the most significantly enriched pathways, ECM and ECM associated, we performed cluster analysis of the data from six donors following bromodomain inhibitor treatment. The heatmap in Fig. 3*E* clearly identifies that each donor falls consistently within each treatment group within the dendrogram, with JQ1 treatment resulting in more potent inhibition than CBP30. We confirmed several of the most significant hits from transcriptomic data using siRNA-mediated knockdown of CREBBP, EP300, and BRD4, also including combined CREBBP/EP300 knockdown to represent CBP30 targeting both bromodomains (*SI Appendix*, Fig. S6 *A*–*L*).

Having established the profound transcriptional effect of bromodomain inhibition on profibrotic gene expression, we extended our analysis to include the effects on global protein expression following bromodomain inhibition. Shotgun proteome analysis identified a limited number of significantly regulated proteins (*SI Appendix*, Table S5), most notable being the three collagen VI (collagen VIα1/2/3) isoforms in CBP30-treated cells (Dataset S4). We integrated the pathways generated by the transcriptomic dataset of CBP30-treated myofibroblasts with the proteome analysis to systematically explore the broader molecular landscape directed by CBP30 in myofibroblasts. This enabled a comprehensive CBP30 pathway analysis, which was found to be dominated by pathways associated with extracellular matrix organization (Fig. 3*F*). Focused correlation analysis of individual gene/protein expression levels showed collagen VIα1/2/3 and fibronectin were core targets driving the enriched ECM pathways (Fig. 3*G*). To validate our findings, we performed Western blotting using the bromodomain inhibitors on steady-state expression of key ECM components. We observed marked inhibition of collagen Iα1, collagen VIα1, EDA-FN, and α-SMA after 6 d of treatment with the bromodomain inhibitors (Fig. 3*H*).

### Collagen VI Drives a Profibrotic Phenotype in Myofibroblasts.

**Fig. 4.**
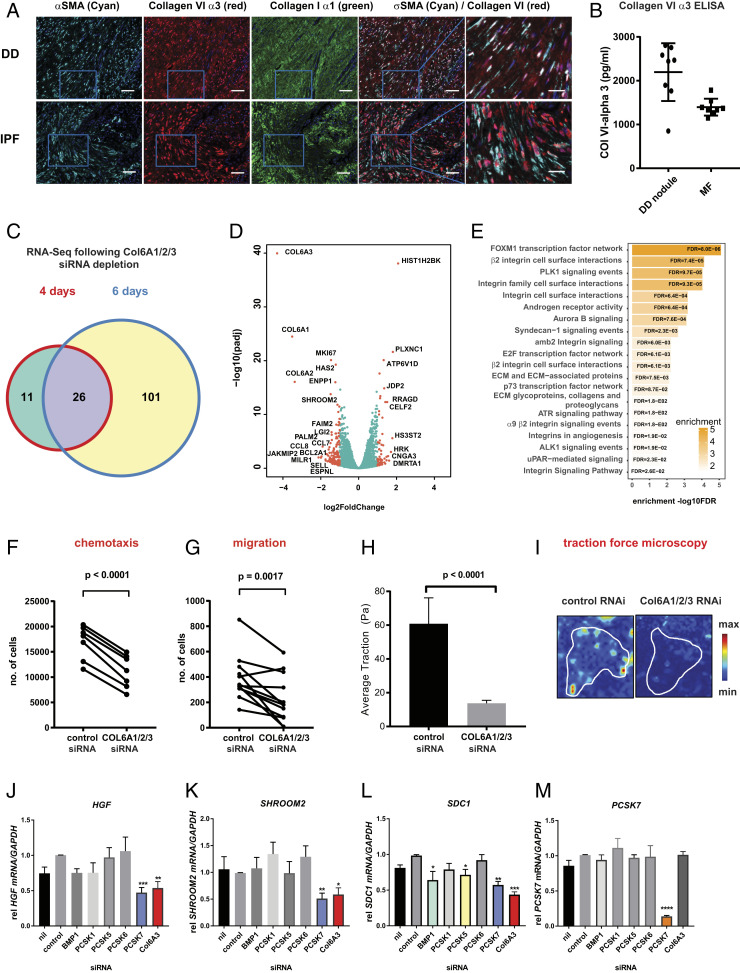
Collagen VI drives a profibrotic phenotype in myofibroblasts. (*A*) Confocal microscopy after immunostaining using anti-αSMA, anti-collagen VI α3, or collagen I α1-specific antibodies in either (*Top*) DD nodule or IPF (*Bottom*) sections; representative of three donors, three slides per donor. (Scale bar, 25 or 50 µM for higher magnification slides.) (*B*) Freshly dissociated DD nodule cells or passaged (p2) DD myofibroblasts were cultured for 3 d and secreted collagen VI α3 levels were detected by ELISA. (*C*) Venn diagram showing the number of genes with significant differential expression (log2 FC > 1.5, FDR < 0.05) following either nontargeting control siRNA (20 nM) or *COL6A1/2/3* siRNA (20 nM)-mediated depletion over 4 or 6 d. (*D*) Volcano plots of differentially expressed genes, at day 6; data are generated from six donors. (*E*) Gene enrichment analysis of canonical pathways of significantly differently expressed genes at day 6. (*F*) THP-1 chemotaxis to myofibroblast-conditioned media generated from control siRNA (20 nM) or *COL6A1/2/3* siRNA-treated cells for 6 d; migrated cells were quantified by Hoechst 3342 fluorescent stain for 1 h using the Celigo Imaging Cytometer. (*G*) Scratch assay measuring migration of myofibroblasts following *COL6A1/2/3* depletion; migrated cells were quantified by staining with calcein fluorescent stain for 1 h using the Celigo Imaging Cytometer in eight donors. *P* value was determined by paired sample *t* test in eight donors. (*H*) Effect of *COL6A1/2/3* depletion as measured by traction force microscopy; cells were detached after 6 d in presence of 20 nM siRNA. Color scale in stiffness maps indicates shear modulus in kilopascals. Representative (*I*) traction vector maps, average traction is depicted (mean ± SEM; *n* = 4 independent experiments with a minimum of 20 independent cells per condition; **P < 0.01 and *P ≤ 0.05 versus controls). Six-day siRNA-mediated PCSK (20 nM) depletion in DD myofibroblasts inhibits (*J*) *HGF*, (*K*) *SHROOM2*, (*L*) *SDC1*, and (*M*) *PCSK7* gene expression as measured by Taqman PCR using the ∆∆Ct method normalized to GAPDH; mean ± SEM from six donors. *P* value was determined by one-sample *t* test to normalized control (nontargeting oligo) sample of value of 1. *P ≤ 0.05, **P < 0.01, ***P < 0.001.

Our multiomics profiling converged on collagen VI as a principal downstream target regulated by CBP/EP300. Immunostaining confirmed expression of the α3 chain of collagen VI throughout the nodules of DD, colocalizing with α-SMA positive myofibroblast foci (Fig. 4*A*). Similarly, collagen VI α3 was also associated with α-SMA positive myofibroblasts localized to the fibrotic foci of samples from patients with IPF (*SI Appendix*, Fig. S7). We also confirmed the presence of abundant levels of the full-length α3 chain of collagen VI in tissue culture supernatants of freshly isolated Dupuytren’s nodular cells and passaged MFs (Fig. 4*B*).

As collagen VI was highly associated with the myofibroblast populations in human fibrosis, we further explored its function. We performed RNA-Seq on DD myofibroblasts treated with *COL6A1*/*A2*/*A3* siRNA for either 4 or 6 d (Fig. 4*C*) (37). The differentially expressed genes are displayed as a volcano plot (Fig. 4*D*). This revealed a discrete number of differentially expressed genes, highlighting that more genes were targeted following longer exposure to *COL6* depletion. These genes included *HGF*, *SDC1*, *CCL2*, *CCL7*, *ADAMT4/8*, and *HAS2* (Dataset S5). These differentially expressed genes were associated with ECM remodeling, FOXM1 transcription network, integrin signaling, Syndecan-1, and the androgen receptor activity pathways (Fig. 4*E* and Dataset S6). This latter finding is of particular interest, given the male predominance of Dupuytren’s disease in younger age groups (38). Syndecan-1 has been shown to regulate ECM fiber alignment and the activities of several integrins, including αvβ3, αvβ5, and α2β1 which control stromal cell migration (39). When comparing our EP300 ChIP-Seq dataset, we found 45% of the differentially regulated genes (log2 fold change [FC] > 1) were also enriched for EP300 binding sites. Notably, we found siRNA depletion of *COL6A3* alone was sufficient to significantly suppress the expression of multiple target genes reported in the RNA-Seq (*SI Appendix*, Fig. S8), suggesting this may be the dominant isoform driving the profibrotic function of collagen VI.

We asked whether the downstream targets of collagen VI are functional in human myofibroblasts. As *COL6A1/2/3* depletion markedly reduces expression of multiple chemokines, we confirmed that *COL6A1/2/3* siRNA treatment significantly inhibited CCL2 and CCL7 protein secretion by myofibroblasts (*SI Appendix*, Fig. S9). We confirmed that *COL6A1/2/3* depletion in myofibroblasts significantly reduced chemotaxis of the myeloid cell line THP-1 toward myofibroblast conditioned media, suggesting collagen VI may play an important role in promoting immune cell recruitment in localized fibrosis (Fig. 4*F*). As the *COL6A1/2/3* targets *HGF* and *SDC1* are known mediators of cell migration (39), we next sought to determine whether collagen VI regulates myofibroblast migration using an in vitro wound healing assay. We found that myofibroblast migration was significantly impaired following *COL6A1/2/3* depletion (Fig. 4*G*). Finally, as many genes regulated by collagen VI are associated with ECM interaction and integrin pathways, we investigated the effect of *COL6A1/2/3* depletion on contractility of myofibroblasts on collagen-coated hydrogels using traction force microscopy. We found that *COL6A1/2/*3 depletion resulted in a pronounced reduction of contraction (Fig. 4 *H* and *I*). Collectively, these data demonstrate the collagen VI pathway, regulated by EP300, orchestrates multiple important profibrotic functions, including chemoattraction, migration, and contractility of human myofibroblasts.

Proprotein convertases process and modulate proteins within the secretory compartment, at the plasma membrane and in the extracellular space (40) and, along with the procollagen C-proteinase BMP-1, have recently been described as key regulators in the proteolytic cleavage of collagen VI α3 (41). Analyzing our RNA-Seq datasets we identified all of the proprotein convertase subtilisin/kexin (PCSKs) expressed by the DD myofibroblasts and systematically depleted them to determine their importance in the regulation of the COL6A3-dependent gene expression. We found that several genes identified from our gene enrichment pathway analysis following the *COL6A1/2/3* RNA-Seq analysis within the Syndecan-1 pathway (*HGF* and *SDC1*) were also regulated by PCSK7 to a level similar to that observed by *COL6A3* depletion alone (Figs. 4 *J*–*M*). PCSK7 depletion also regulated the chemokine *CCL26* and its family member PCSK1 inhibited *ADAMTS8* (*SI Appendix*, Fig. S10).

## Discussion

Clinical trials in human fibrosis have repeatedly failed, probably due to the poor predictive accuracy of targets identified using murine models of fibrosis. DD represents an important platform to study mechanisms of human fibrosis because of the ready availability of tissue from patients undergoing surgery as a therapeutic intervention. We combined multiomic profiling with functional validation using these well-characterized patient samples and identified collagen VI as a major regulator of the myofibroblast phenotype.

Our genetics-led network connectivity algorithm (Pi) proposed CREBBP/EP300 as key epigenetic regulators of the myofibroblast phenotype in DD. Screening of a panel of high-quality epigenetic probes highlighted bromodomain inhibitors as potent regulators of profibrotic gene expression in DD myofibroblasts. While relying on chemical probes alone has pitfalls due to potential off-target effects, here we provide multiple lines of evidence of supporting a direct role of CBP/EP300 in profibrotic gene regulation. First, two structurally distinct bromodomain inhibitors CBP30 and I-CBP112, and the EP300 HAT inhibitor A485, display similar activity profiles in suppressing gene expression, with an IC_50_ of 1 μM, consistent with reported activity on the CBP-bromodomain (42, 43). Second, siRNA-mediated suppression of CBP/EP300 also confirmed a role of these epigenetic modifiers in the regulation of numerous genes identified in the RNA-Seq dataset. Critically, using paired epigenetic and transcriptomic profiling, we provide a mechanistic insight and confirm bromodomain inhibition directly blocks gene transcription through inhibiting the recruitment of CREBBP/EP300 to their binding sites, thereby reducing levels of H3K27ac at the *ACTA2* and *COL1A1* loci.

H3K27ac and H3K4me1 occupancy are chromatin features used to identify cell-type-specific enhancers (44, 45). Significantly, our data show a high degree of genomic co-occupancy of EP300 with H3K27Ac and H3K4me1, indicating that super enhancer sites are indeed regulating coordinated transcriptional control of the highly enriched ECM pathways dominating the EP300 pathway analysis in myofibroblasts. Interestingly, EP300 showed a distinct binding profile closely localizing with 8 of the 22 most significant SNPs associated with Dupuytren’s disease (12). Since both H3K27ac and EP300 (32, 46) demark the architecture of super enhancers in all cell types, this warrants further investigation using coactivators such as MED1 and other master transcription factors. Of particular note is the SFRP4 variant, as this was shown to be the most strongly associated variant (rs16879765) in our recent DD GWAS study (12) and we have shown here that SFRP4 is regulated by FOSL1 depletion. Furthermore, the study by Ng et al. (12) suggested that a subtle imbalance of WNT signaling contributes to the fibrotic phenotype, allowing an increase in WNT3A signaling through the noncanonical pathway. Wnt/β-catenin signaling has been shown to activate Fra1 expression, and Fra1 has been shown to play a role in epithelial–mesenchymal transition (EMT) activated by Wnt/β-catenin signal pathway (47).

Our epigenetic profiling and motif analysis revealed several putative EP300 coregulators of gene transcription, including Fra1, RUNX1, and TEAD4. Functional analysis of these transcription factors demonstrated discrete functional properties of each in myofibroblasts. Fra1 (*FOSL1*) has previously been reported as a negative regulator of AP-1 (48–50) and its depletion enhanced bleomycin-induced lung fibrosis. Our data confirm Fra-1 acts as a transcriptional repressor of *CXCL8*, *IL6*, and *CTGF* in DD myofibroblasts but conversely acts to positively regulate *SPFR4*, *CCL26*, and *COL6A3* expression, suggesting a highly complex level of regulation of the transcriptional machinery at these genes. TEAD factors act as mediators of the Hippo signaling pathway interacting with the YAP and WWTR1 (TAZ) transcriptional coactivators (51) and EP300 has recently been shown to activate YAP/TAZ (52). We observed TEAD4 depletion resulted in decreased expression of two key chemokines *CCL2* and *CXCL8* (IL-8), important recruiters of monocytes and neutrophils. Depletion of RUNX1 highlighted a role of this transcription factor in the regulation of two key ECM regulators, fibronectin-1 (*FN-1*) and Hyaluronan Synthase 2 (*HAS2*). Together, these data describe a transcriptional network downstream of EP300 that orchestrates diverse cellular profibrotic functions in myofibroblasts associated with pathogenicity, including enhanced matrix remodeling, development of a contractile phenotype, and immunomodulatory properties, thereby providing a framework by which these transcription factors link EP300 gene regulation with the profibrotic phenotype typically associated with myofibroblasts.

Collagen VI is an interstitial collagen that functions as a structural component of the matrix in addition to influencing cell adhesion. Levels are elevated in fibrotic conditions such as IPF (53), chronic liver disease (54), and chronic kidney disease (55). Fragments from the C-terminal part of the α3 chain have been shown to have signaling effects, including profibrotic features and macrophage chemoattractant properties. One such fragment PRO-COL6/endotrophin is now commonly used as a biomarker in cancer (56) and fibrotic conditions (57, 58) and has been able to distinguish individuals with IPF who have progressive disease from those with more stable disease (59). We confirmed the presence of high levels of collagen VIα3 in supernatants from freshly isolated Dupuytren’s nodules and in cultured myofibroblasts. Our immunofluorescence studies highlighted that it is highly expressed in both Dupuytren’s nodules and IPF patient samples, localizing closely with α-SMA positive myofibroblasts regions. It is interesting to note that whereas collagen VIα3 was widely distributed in DD nodules, in the IPF samples a more discrete cellular localization was observed when compared to collagen Iα1, suggesting discrete functions as dictated by the local cellular–stromal architecture. Alternatively, it may reflect differing disease stages as IPF is usually diagnosed late. Furthermore, we show that collagen VI regulates expression of the chemokines *CCL2*, *CCL7*, and *CCL26*, in accordance with previous data suggesting a potential role of endotrophin (ETP) in chemotaxis (60), and we confirmed a role of collagen VI in myofibroblast-induced chemotaxis of THP1 cells. Previously (4), we identified macrophages and mast cells as the predominant source of TNF within Dupuytren’s nodule cultures and demonstrated that macrophage-derived TNF plays a key role in promoting the fibrotic phenotype in Dupuytren’s disease (4). Excessive production of collagen VI may contribute to the chronicity of the disease by promoting the recruitment of monocytic cells to the site of this disease and probably plays a similar role in other fibrotic disorders (61). A central function of myofibroblasts in both health and disease is the generation of traction force (62), which plays a key role in remodeling the matrix and also modulates the activities of the embedded stromal cells (63, 64). Importantly for disease management, we also demonstrated that collagen VI regulates myofibroblast contraction.

Mutations in collagen VI genes is associated with Bethlem myopathy (BM) and Ullrich congenital muscular dystrophy (UCMD) (65). A mouse model in which the *Col6a1* gene was inactivated has identified key functions of *Col6a1*, including autophagic dysfunction, skeletal, heart, and tendon defects (66). COL6A3 protein deficiency in mice leads to muscle and tendon defects similar to those seen in human collagen VI congenital muscular dystrophy (67). Therefore, directly targeting collagen VI is likely to be deleterious. The C5 region of the α3 chain of collagen VI has been shown to undergo proteolysis in a tissue-specific manner by the proprotein convertase furin/PCSK3 and BMP1 (68), generating diverse fragment sizes depending on the tissue source (41). However, free endotrophin was shown to be scarce under physiological conditions, suggesting that the proteolysis of collagen VI is tightly regulated. Hence targeting the collagen VI proteolysis cleavage machinery could represent a more tractable approach. Analysis of our RNA-Seq datasets revealed that PCSK3 is not expressed in the DD myofibroblasts, but we were able to detect mRNA expression of PCSK1, PCSK5, PCSK6, and PCSK7, with PCSK7 showing the highest basal level of gene expression. Our siRNA studies identified PCSK7 as a potential protease regulating collagen VIα3 cleavage. Depletion of PCSK7 suppressed gene expression of a subset of the genes from the Syndecan-1 signaling pathway, a key pathway regulating stromal cell migration and ECM fiber alignment (39); this was also regulated by *COL6A3* depletion. Our future work will explore the role of PCSK7 in collagen VI proteolysis and work is underway to identify any PRO-C6 fragments in our model. It is noteworthy that *PCSK7* missense variants identified in GWAS studies are associated with liver fibrosis (69). More recently a *PCSK7* variant has been shown to lead to increased intracellular PCSK7 expression and secretion from hepatocytes, potentially linking dyslipidemia with a tendency to more severe liver damage in high risk individuals (70). Given that PCSK7 can be secreted as well as localized on the plasma membrane, this raises the potential for therapeutic development of monoclonal-based PCSK7 inhibitors. The translational potential of the wider family of proprotein convertases has already been realized as there are two FDA-approved drugs, evolocumab and alirocumab, targeting the plasma protein PCSK9 for the treatment of familial hypercholesterolemia and clinical atherosclerotic cardiovascular disease (71). Further work is needed to explore the tissue-specific nature of PCSK7 expression and a wider understanding of its physiological role.

A limitation of this study is the absence of appropriate control tissue. Dupuytren’s disease is restricted to certain fibers of the palmar fascia. Previous studies used cells from the fascia in the region of the carpal tunnel or the transverse carpal ligament from affected or normal individuals or uninvolved transverse palmar fibers from patients with Dupuytren’s disease as controls (72). However, this approach has significant limitations. The palmar fascia over the carpal tunnel is rarely affected by Dupuytren’s disease and the transverse carpal ligament is always unaffected; hence, it is possible that the constituent cells are inherently different. Furthermore, normal palmar fascia is sparsely populated by cells and hence to obtain adequate numbers requires repeating passaging of cells in vitro (73). Furthermore, we and others have shown that at passage 5 the phenotypes of myofibroblasts and normal human dermal fibroblasts tend to merge (74).

**Fig. 5.**
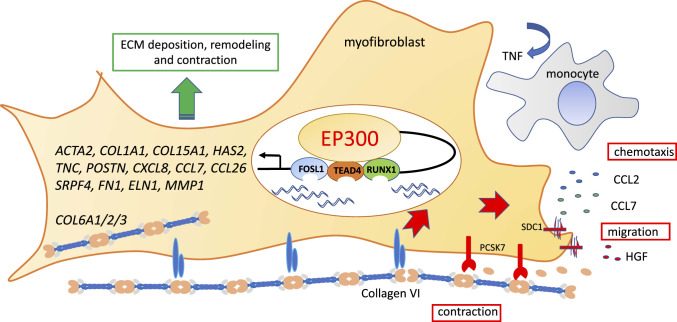
Schematic illustrating the epigenetic control by EP300 of the profibrotic phenotype of myofibroblasts in Dupuytren’s disease. Persistent activation of EP300 acetylates histones and transcription factors to promote extracellular matrix production and myofibroblast contractility. Collagen VI, a key target of EP300, plays a dominant role in regulating contraction. Proteolytic cleavage by PCSK7 generates small bioactive collagen VI fragments which control recruitment of immune cells by chemokine production, thereby perpetuating the cycle of chronic inflammation and fibrosis by the secretion of cytokines such as TNF.

In summary, by exploiting the availability of samples from patients with DD and a combination of epigenetic and transcriptional profiling studies, we defined EP300 as an important regulator of human fibrosis that directs diverse cellular processes in myofibroblasts. There are supporting data showing that pharmacological inhibition of EP300 is effective in murine models of pulmonary fibrosis (75, 76). However, the many targets of epigenetic regulators suggest their long-term global inhibition may result in off-target effects. Here we reveal that collagen VI is a downstream target of EP300 and is widely expressed in DD nodules and IPF lung tissue and mediates an important role in regulating ECM production, chemotaxis, and contractility as shown in Fig. 5. Crucially, our data with EP300 regulation identify collagen VI and PCSK7 as previously unidentified therapeutic targets. The important translational potential of our findings is underpinned by using primary human samples exclusively, including Dupuytren’s disease and IPF, diseases where therapeutic options are limited and patient outcomes are currently suboptimal.

## Methods

In brief, screening the effects of a panel of epigenetic inhibitors upon *ACTA2*, *COL1A1*, *COL3A1* and *TGFB1* gene expression was performed using primary human myofibroblasts isolated from DD patients by Taqman PCR. Inhibition of acetylation at the promotors of *ACTA2* and *COL1A1* by the EP300 probe SGC-CBP30 (CBP30) was shown using ChIP PCR. Inhibition of myofibroblast contraction was assessed using traction force microscopy (23). Evidence of direct recruitment of EP300 to a profibrotic gene landscape was shown using ChIP-Seq. Transcriptional profiling of CBP30 in myofibroblasts was performed using RNA-Seq and hits confirmed using siRNA of CREBBP/EP300 depletion studies. The effects of CBP30 on global protein expression in DD myofibroblasts was determined using shotgun proteomic profiling. The role of collagen VI in controlling profibrotic gene expression was confirmed by siRNA-mediated *COL6A1/2/3* depletion followed by RNA-Seq; subsequent roles in contraction, migration, and wound healing were confirmed using traction force microscopy, chemotaxis, and scratch assays, respectively. The methodology is described in detail in *SI Appendix*.

## Supplementary Material

Supplementary File

Supplementary File

Supplementary File

Supplementary File

Supplementary File

Supplementary File

Supplementary File

## Data Availability

RNA-Seq and ChIP-Seq datasets are deposited within the NCBI SRA database under accession numbers PRJNA625874, PRJNA624331, and PRJNA624119, respectively.
