# Supplementary material for: Identifying collagen VI as a target of fibrotic diseases regulated by CREBBP/EP300
Source: Proc Natl Acad Sci U S A. 2020 Aug 5;117(34):20753–63. doi: 10.1073/pnas.2004281117 (PMC7456151; doi:10.1073/pnas.2004281117)
Supplement: Supplementary File [file pnas.2004281117.sapp.pdf]

Identifying collagen VI as a target of fibrotic diseases regulated by CREBBP/EP300

Authors: Lynn M. Williams[1]†, Fiona E. McCann[1†], Marisa A. Cabrita[1], Thomas Layton[1], Adam Cribbs[2], Bogdan Knezevic[3], Hai Fang[3], Julian Knight[3,] Mingjun Zhang[4], Roman Fischer[5], Sarah Bonham[5], Leenart M. Steenbeek[6], Nan Yang[1], Manu Sood[7], Chris Bainbridge[8], David Warwick[9], Lorraine Harry[10], Dominique Davidson[11], Weilin Xie[4], Michael Sundström[12], Marc Feldmann[1], Jagdeep Nanchahal[1*.]

Content

1. *SI Appendix,* **Supplementary Materials and Methods**
2. *SI Appendix,* **References**
3. *SI Appendix,* **Tables 5**
4. *SI Appendix,* **Figures 10**

www.pnas.org/cgi/doi/10.1073/pnas.2004281117

1. SI Appendix, Supplementary Materials and Methods

Dupuytren's Tissue Collection

Nodular tissue was obtained from patients with Dupuytren's disease undergoing dermofasciectomy as previously described (1). Informed written consent was obtained through approval of the regional ethics committee (REC 07/H0706/81), in accordance with the Declaration of Helsinki**.**

Cell culture

Tissue samples were dissected into small pieces and digested in Dulbecco's Modified Eagle Medium (DMEM) (Gibco,UK) with type I collagenase 4mg/ml (Worthington Biochemical Corporation) + DNase I (Roche Diagnostics) for up to 3 hours at 37 °C. Digested tissue fragments were filtered through a 70-μm cell strainer, and the cell suspension was centrifuged at 1500 rpm for 10mins. Isolated cells were cultured in DMEM with 5% FBS and 1% penicillin–streptomycin and used until passage 2.

Compound screening

Using a focused library of small-molecule inhibitors targeting epigenetic modifications, provided by Structural Genomics Consortium, Oxford (Table S2), we assessed their effect on steady state mRNA expression levels of *ACTA2, COL1A1,* and *TGFB1* in passage 2 DD myofibroblasts. All experiments were harvested after 3 or 7 days in culture and performed in cells from 5 donors, using compound doses between 0.1-10μM. Cell viability was assessed after 3 or 7 days using the MTS assay (Promega).

Transfections for siRNA

Cells were seeded at 2 x 10^4/24 well plate and the following day transfected with siRNA against BRD4 (s23901), CREBBP (s3595), EP300 (s4695) or non-targeting control oligo at 1-20nM (Life Technologies). All transfections were performed with Dharmafect 1 (GE Healthcare) and Optimem™ (Life Technologies) according to the manufacturer's instructions. Media was replaced 2 hrs later with 5% FBS phenol red free DMEM (Gibco). Experiments were performed either 3-6 days post transfection.

Antibodies

Antibodies used in immunoblotting; anti-αSMA (Sigma A5228), anti-BRD4 (Abcam 128874), anti-CREBBP (Cell Signalling 7425), anti-EP300 (Millipore 05-257), anti-collagen alpha-1(I) chain (Millipore ABT257), anti-collagen alpha-1(VI) chain (Abcam 182744), anti-collagen alpha-3(VI) chain (Millipore MAB1944), Fibronectin (ED-A, Abcam 632800), Fibroectin-ED-B (Abcam 154210), anti-Lamin B1 (Santa Cruz 20662) and anti-Lamin A/C (BD Biosciences 612163). Antibodies used in ChIP experiments; rabbit Ig (Diagenode C15410206), anti-BRD4 (Cell Signallin E2A7X), anti-EP300 (Millipore 05-257), anti-CREBBP (Abcam ab2832) (Diagednoe C15200211), anti-H3K27ac (Abcam ab177178) and anti-H3K4me1 (Cell Signaling D1A9).

Measurement of mRNA

RNA was extracted using Quick-RNA MiniPrep kit (Zymo) and cDNA was generated using the RNA-Ct kit (Life Technologies). Gene expression was determined by quantitative PCR on a ViiA™7 using Taqman™ Fast Advanced Mastermix (Life Technologies) and Assay-On-Demand premixed Taqman™ probe master mixes (Life Technologies, Table S4). The relative gene expression was calculated using the ΔΔCt method with the *GAPDH* gene for normalization of mRNA levels.

RNA isolation and RNA-seq library preparation

RNA was isolated from myofibroblats using the Quick-RNA MiniPrep kit (Zymo) according to the manufacturer's protocol. The quality of the RNA samples was verified by electrophoresis on 2200 TapeStation (Agilent). The RIN scores for all samples were in the range of 7.5–9.5.RNA-seq libraries were prepared using the NEBNext® Ultra™ RNA library prep kit for Illumina® using NEB indexes, according to the manufacturer's protocol. The concentration of each library was determined using Agilent TapeStation. The resulting libraries were sequenced on a NextSeq 500 platform (Illumina) using a paired-end run 2×80 bp, to an average depth of 20×10^6 paired-end reads/sample. The concentration of each library was determined using Agilent 2200 TapeStation.

Chromatin immunoprecipitation (ChIP) assay/ChIP-Seq

For ChIP experiments nuclear lysates from 2×10^6 myofibroblasts were fixed for 10 minutes in 1% formaldehyde and were isolated as described previously (2) and subjected to 6 cycles of sonication using the Biorupter Pico system (Diagenode). Each lysate was immunoprecipitated using 2.5-5 µg of the relevant antibodies. Real-time PCR was performed on fragmented DNA using specific primers for the *ACTA2* or *COL1A1* (*ACTA2*-F 5'- gaaggcttggcgtgtttatc-3', *ACTA2*-

R, 5’- cttgcttcccaaacaaggag-3’, *COL1A1*-F, 5’- gccagtcgtcggagcaga-3’, *COL1A1*-R, 5’-ctcgacttggccttcctctt-3) using SYBER green Power Master mix (Life Technologies). ChiP-Seq was performed using 2 biological replicates from 2 donors. Nuclear lysates from 10 x 10^6 myofibroblasts were fixed in 1% formaldehyde for 15mins prior to quenching. For histone marks H3K27ac, H3K4me1 and BRD4 chromatin was precleared with protein G Dynabeads (Life Technologies) prior to an overnight incubation at 4°C with 5-10ug of protein G pre-coupled antibody. DNA was purified using Qiagen PCR purification kit. For EP300 ChIP was performed with 25 x 10^6 myofibroblasts using iDeal transcription factor kit (Diagenode) as per manufacturer’s protocol, with an additional 30 minute fixation step using ChIP cross-ling Gold (Diagenode), followed by a further conventional fixation step of 10 minutes with 1 % formaldehyde. Purified DNA was used for library preparation using a NEBNext ® Ultra ™ II sample preparation kit (NEB) according to the manufacturer's recommendations. The samples were multiplexed, quantified using a Bioanalyser (Aglient), and sequenced on a NextSeq 500 (Illumina) platform (paired-end, 2 × 41 bp). Sequencing depth was in excess of 20 million reads/sample, suggesting sufficient coverage.

Processing of next-generation sequencing data

A computational pipeline was written calling scripts from the CGAT toolkit to analyse the next generation sequencing data https://github.com/cgat-developers/cgat-flow (3). For RNA-seq experiments, sequencing reads were mapped to the reference human genome sequence (GRCh37 (hg38) assembly) using HISAT v0.1.6 (4). To count the reads mapped to individual genes, the program featureCounts v1.4.6 (5) was used. Only uniquely mapped reads were used in the counting step. The counts table that was generated was then used for differential expression analysis. Differential gene expression analysis was performed with DESeq2 v1.12.3 within the R

statistical framework v3.3.0 (6). To define differentially expressed genes, a threshold of >2-fold change, and a false discovery rate of <0.05 were used. For analysis of ChIP-seq, Bowtie2 software v0.12.5 was used to align the reads to the human hg19 reference genome (7). Reads were only considered that were uniquely aligned to the genome with up to two mismatches. The peak calling step used MACS2 using the broad option and for each condition the corresponding input was used. Homer tag directories were then produced using the raw read function, and coverage plots around the TSS were plotted in R. MACS software (v1.4.2) or SICER (v1.1) was used to identify enrichment of intervals of EP300, BRD4 H3K4me3 and H3K27ac following ChIP-seq. Sequencing of the whole cell extract was performed to determine the background model when analysing ChIP-seq. Enrichment of reads around the TSS and enhancer sites was calculated and plotted across the genome using the ngs.plot.r package (8). For visualization as a University of California Santa Cruz Genome Browser track, the bam files generated from Bowtie were converted to BigWig files using bam2wiggle (9). Pathway enrichment analysis was performed using XGR (10).

Data Availability

RNA-seq, and ChIP-seq datasets are deposited within the SRA database under accession numbers PRJNA625874, PRJNA624331 and PRJNA624119 respectively.

Immunofluorescence and confocal microscopy

Myofibroblasts were grown on poly-L lysine (Sigma) coated glass coverslips and stained for F-actin as previously described (1). Fluorescent images were obtained with a LSM710 confocal microscope (Leica Microsystems). Circularity: $4\pi \times ([\text{Area}] / [\text{Perimeter}]^2)$, with a value

of 1.0 indicating a perfect circle), Feret and Perimeter calculations were analyzed and processed with ImageJ.

Mass spectrometry analysis and protein identification

DD myofibroblasts were treated with wither DMSO, JQ1 (0.5µM) or CBP30 (2.5µM) for 3 days. Cell pellets were lysed in 25 µl of lysis buffer, consisting of radioimmunoprecipitation assay (RIPA) buffer (Sigma) with 4% NP-40 (IPEGAL, Sigma). After thawing, sample preparation involved an in-solution digestion protocol followed by a purification step. Briefly, after reduction and alkylation of cysteine residues, protein precipitation was undertaken via methanol/chloroform extraction (REF: https://www.ncbi.nlm.nih.gov/pubmed/6731838). After this, we carried out an overnight digestion in 0.2µg/ml Trypsin (Promega) at 37°C and purified the resulting peptides by reversed-phase extraction. Peptides were analyzed with a nLC-MS/MS platform consisting of a Dionex Ultimate 3000 and Orbitrap Fusion Lumos mass spectrometer (Thermo Scientific). Label-free quantification of proteins was performed using Progenesis QI for Proteomics (version 4.1, Waters) and proteins were identified using MASCOT (Matrix Science) by searching against the Swissprot human database (Feb. 2018). The Benjamini–Hochberg multiple testing correction was used to estimate the FDR.

Traction force microscopy

Polyacrylamide (PAA) (Bio-Rad, CA) hydrogels used in traction force microscopy were prepared as previously described (4). The Young's modulus of the PAA gels were 2.55±0.5 KPa. Bead displacement was tracked with an ImageJ PIV plugin (11) (12) and cellular forces reconstructed using a FTTC algorithm also implemented in ImageJ. Plots were generated in MATLAB (Mathworks).

.

Chemotaxis Assay

5 x 10^5 THP-1 cells (ATCC) were allowed to adhere for 2 hrs to a Corning Transwell® plate with 8µM pore in 1% FBS DMEM, the reservoir plate was then replaced with conditioned media generated from DD myofibroblasts treated with either control non-targeting Silencer Select™ siRNA (20nM) or a combination of *COL6A1/2/3* siRNA (8.3nM each) (Life Technologies, UK). For the last 3 days of the assay, the media was replaced with 1% FBS, DMEM. Supernatants harvested, filtered and used neat, alongside CCL2 (2 ng/ml) (Peprotech, London UK) as a positive control. Cells were allowed to migrate for 6 hrs, the number of migrated cells were counted in the reservoir plate following the addition of Hoechst 3342 Fluorescent Stain (Life Technologies) at 0.1µg/ml for 1 hr using the Celigo Imaging Cytometer, Nexcelom Bioscience (Lawrence, MA).

Scratch Wound Assay

5 x 10^3 DD myofibroblasts were allowed to adhere for 24 hrs to a collagen coated (0.1μg/ml) 96 well tissue culture plate. Cells were visualised by treating with Calcein-AM for 1 hr prior making a scratch using an IncuCyte® woundmaker. Cells were imaged using a Celigo Imaging Cytometer, Nexcelom Bioscience (Lawrence, MA), and then imaged 24 hrs later and whole-well images were acquired using the Celigo Wound Healing application which reports the wound healing confluence and cell count for each well over time within the defined analysis area.

Immunofluorescent Microscopy

Multiplex-immunofluorescent (IF) staining of COL1A1/αSMA/COL6A3 was performed on Leica Bond-Rx Autostainer (Leica Microsystems) with Opal 7-Color Automation IHC Kit (PerkinElmer, NEL821001KT). FFPE tissue sections (4 μm) were antigen-unmasked with epitope retrieval solution 1 (Leica, AR9961) for 20 minutes at 100°C, blocked with PKI blocking buffer for 5 minutes. Anti-Collagen I α1 antibody (Abcam, ab138492, 5ug/ml) was applied for 30 minutes at room temperature, followed by Opal Polymer HRP for 10 minutes, and Opal 540 dye for 10 minutes. The tissue sections were then heated again in epitope retrieval solution 1 for 20 minutes at 95°C to remove excessive unbounded antibody before they were stained for the next antibody. The similar staining cycle was repeated for anti-αSMA antibody (Sigma A5228, 1:15K) with Opal 620 dye, and anti-Collagen α3 antibody (Sigma, HPA010080, 2.8ug/ml) with Opal 690 dye sequentially. Finally the slides were cover-slipped with Prolong gold anti-fade reagent with DAPI (Invitrogen, P36935), and images were taken with Vectra Polaris (PerkinElmer) using Phenochart (version 1.0.10) and inForm (version 2.4.4) softwares.

ELISA

The concentration of collagen 6 $\alpha 3$, CCL2 and CCL7 in cell supernatants was determined by ELISA (BD Biosciences, Oxford UK and R&D systems, Oxford, UK respectively) according to the manufacturer's instructions.

***2. SI Appendix,* References**

1. L. S. Verjee *et al.*, Unraveling the signaling pathways promoting fibrosis in Dupuytren's disease reveals TNF as a therapeutic target. *Proc Natl Acad Sci U S A* **110**, E928-937 (2013).
2. T. Smallie *et al.*, IL-10 inhibits transcription elongation of the human TNF gene in primary macrophages. *J Exp Med* **207**, 2081-2088 (2010).
3. A. P. Cribbs *et al.*, CGAT-core: a python framework for building scalable, reproducible computational biology workflows. *F1000Research* (2019).
4. D. Kim, B. Langmead, S. L. Salzberg, HISAT: a fast spliced aligner with low memory requirements. *Nat Methods* **12**, 357-360 (2015).
5. Y. Liao, G. K. Smyth, W. Shi, featureCounts: an efficient general purpose program for assigning sequence reads to genomic features. *Bioinformatics* **30**, 923-930 (2014).
6. M. I. Love, W. Huber, S. Anders, Moderated estimation of fold change and dispersion for RNA-seq data with DESeq2. *Genome Biol* **15**, 550 (2014).
7. B. Langmead, C. Trapnell, M. Pop, S. L. Salzberg, Ultrafast and memory-efficient alignment of short DNA sequences to the human genome. *Genome Biol* **10**, R25 (2009).

8. L. Shen, N. Shao, X. Liu, E. Nestler, ngs.plot: Quick mining and visualization of next-generation sequencing data by integrating genomic databases. *BMC Genomics* **15**, 284 (2014).

9. D. Sims *et al.*, CGAT: computational genomics analysis toolkit. *Bioinformatics* **30**, 1290-1291 (2014).

10. H. Fang, B. Knezevic, K. L. Burnham, J. C. Knight, XGR software for enhanced interpretation of genomic summary data, illustrated by application to immunological traits. *Genome Med* **8**, 129 (2016).

11. J. L. Martiel *et al.*, Measurement of cell traction forces with ImageJ. *Methods Cell Biol* **125**, 269-287 (2015).

12. Q. Tseng *et al.*, A new micropatterning method of soft substrates reveals that different tumorigenic signals can promote or reduce cell contraction levels. *Lab Chip* **11**, 2231-2240 (2011).

3. SI Appendix, Tables

SI Appendix, Table S1 Pi pathway genes

Name	nAnno[a]	nOverlap[b]	Z score	P value	Adjusted P value	Overlapped genes [c]
FoxO signaling pathway	129	15	7.57	2.70E-08	4.40E-07	MAPK3, AKT1, EGFR, GRB2, CREBBP, EP300, SIRT1, FOXO3, CDK2, GABARAP, MDM2, PLK1, GABARAPL1, GABARAPL2, RBL2
MAPK signalling pathway	247	16	4.91	2.90E-05	0.00023	PRKACA, HSPA1A, HSPA1B, TP53, HSPA8, ATF2, MAPK3, AKT1, TRAF2, RELA, CDC42, EGFR, JUN, MYC, GRB2, NTRK1
ErbB signalling pathway	84	8	4.69	0.00035	0.0018	MAPK3, AKT1, EGFR, JUN, MYC, GRB2, SRC, ERBB2
PI3K-Akt signaling pathway	333	16	3.57	0.0011	0.0043	TP53, ATF2, MAPK3, AKT1, RELA, EGFR, MYC, GRB2, FOXO3, CDK2, MDM2, RBL2, YWHAE, YWHAZ, HSP90AA1, HSP90AB1
cAMP signaling pathway	194	11	3.48	0.0023	0.0072	PRKACA, MAPK3, AKT1, RELA, JUN, PPP1CB, PPP1CC, PPP1CA, CREBBP, CFTR, EP300
Wnt signalling pathway	141	9	3.53	0.0027	0.0073	PRKACA, TP53, JUN, MYC, CTNNB1, CREBBP, EP300, RUVBL1, CSNK1A1
HIF-1 signaling pathway	96	7	3.5	0.0041	0.0093	MAPK3, AKT1, RELA, EGFR, ERBB2, CREBBP, EP300
TGF-beta signaling pathway	82	6	3.24	0.0078	0.016	MAPK3, MYC, CREBBP, EP300, E2F4, SP1
Hippo signalling pathway	150	8	2.73	0.014	0.025	MYC, CTNNB1, ACTB, PPP1CB, PPP1CC, PPP1CA, YWHAE, YWHAZ
TNF signalling pathway	108	6	2.46	0.028	0.045	ATF2, MAPK3, AKT1, TRAF2, RELA, JUN

[a]Number of genes annotated to a pathway; [b]Number of genes in a pathway that overlap with the crosstalk gene network; [c]List of overlapped genes.

***SI Appendix,* Table S2. Binding of EP300 at genomic locations of SNPs significantly associated with Dupuytren's Disease**

Chromosome	Position / rsID	SNP discovery P value	Maximal EP300 binding location	Selected nearby genes
1	162672011 rs17433710	9.13×10^{-7}	162508632	DDR2, HSD17B7
7	37973014 rs2598107	1.10×10^{-30}	37970400	SFRP4, EPDR1
7	37989095 rs16879765	7.15×10^{-41}	37970400	SFRP4, EPDR1
7	116892846 rs38904	1.02×10^{-11}	116899830	WNT2
13	44842503 rs9525927	5.8×10^{-6}	4487194	SMIM2, SERP2
14	23312594 rs1042704	8.72×10^{-13}	23320747	MMP14
14	51074461 rs1032466	4.90×10^{-9}	51142673	ATL1, MAP4K5, SAV1
15	56229760 rs1509406	4.03×10^{-6}	56200240	NEDD4
15	68628163 rs2306022	7.59×10^{-6}	68644962	ITGA11

SI Appendix, Table S3. Epigenetic probes used in this study

Probe	Target Protein family	Specific target	Conc. µM	Duration (days)
**A-366**	Histone lysine methyltransferases	G9a/GLP	0.1	7
**UNC0638**	Histone lysine methyltransferases	G9a/GLP	0.1	7
**UNC0642**	Histone lysine methyltransferases	G9a/GLP	0.1	7
**R-PFI-2**	Histone lysine methyltransferases	SETD7	0.1	7
**GSK343**	Histone lysine methyltransferases	EZH2	0.1	7
**UNC1999**	Histone lysine methyltransferases	EZH2	0.1	7
**LLY-507**	Histone lysine methyltransferases	SMYD2	1	7
**BAY-598**	Histone lysine methyltransferases	SMYD2	0.1	7
**A-196**	Histone lysine methyltransferases	SUV420H2	0.1	7
**SGC0946**	Histone lysine methyltransferases	DOT1L	5	7
**MS023**	Protein lysine methyl transferase	PRMT1,3,4,6,8	2.5	7
**SGC707**	Protein lysine methyl transferase	PRMT3	2.5	7
**TP-064**	Protein lysine methyl transferase	PRMT4,6	2.5	7
**MS049**	Protein lysine methyl transferase	PRMT4,6	2.5	7
**GSK591**	Protein lysine methyl transferase	PRMT5	2.5	7
**OICR-9429**	Methyl lysine binder	WDR5	0.1	3
**A-395**	Methyl lysine binder	EED	0.1	3
**UNC1215**	Methyl lysine binder	L3MBTL3	0.1	3
**NVS-CER2-1**	Bromodomains	CERC2	1	3
**LP99**	Bromodomains	BRD7, 9	10	3
**BI-9564**	Bromodomains	BRD7, 9	1	3
**TP472**	Bromodomains	BRD7, 9	0.5	3
**I-BRD9**	Bromodomains	BRD 9	1	3
**BAZ2-ICR**	Bromodomains	BAZ2A/B	10	3
**GSK2801**	Bromodomains	BAZ2A/B	10	3
**Bromosporine BrSp**	Bromodomains	BRD2, BRD3, BRD4, BRDT	5	3
**JQ1**	Bromodomains	BRD2, BRD3, BRD4, BRDT	5	3
**PFI-1**	Bromodomains	BRD2, BRD3, BRD4, BRDT	10	3
**PFI-3**	Bromodomains	SMARCA2/4	10	3
**I-CBP112**	Bromodomains	CREBBP/EP300 BRD	10	3
**SGC-CBP30**	Bromodomains	CREBBP/EP300 BRD	10	3
**NI-57**	Bromodomains	BRPF1	10	3
**PFI-4**	Bromodomains	BRFP1	10	3
**BAY-299**	Bromodomains	BRFP1B/2	10	3
**OF-1**	Bromodomains	BRFP1B/2	10	3
**L-Moses**	Bromodomains	PCAF,GCN5	1	3
**GSK-J4**	Histone demethylase	JMJD3/UTX	5	3
**GSK-LSD1**	Histone demethylase	Lysine specific demethylase 1	10	3
**IOX2**	2OG	2-oxoglutarate oxygenases	10	3

***SI Appendix*, Table S4. Taqman primers and siRNA oligos used in this study**

**Target**	**Taqman primer/probe**	**siRNA**	**Target**	**Taqman primer/probe**	**siRNA**
ACTA2	Hs00426835_g1		*GAPDH*	4352665	
ADAMTS-4	Hs00192708_m1		*HAS2*	Hs00193435_m1	
ADAMTS-8	Hs00199836_m1		*HGF*	Hs00300159_m1	
BMP1	Hs00241807_m1	S2017	*IL6*	Hs99999032_m1	
BCL2A1	Hs0018745_m1		*IL8*	Hs00174103_m1	
BRD4	Hs00293232-m1	s23901	*ITGA10*	Hs01006910_m1	
CCL2	Hs00234140_m1		*JAM2*	HS01022006_m1	
CCL7	Hs00171147_m1		*MMP1*	Hs00899658_m1	
CCL26	Hs00171145_m1		PCSK1	Hs01026107_m1	S10147
CCNB1	Hs01030099-m1		PCSK5	Hs001964400_m1	n323338
Col1A1	Hs00943009_m1		PCSK6	Hs01060079_m1	S224155
Col6A1	Hs00355783-m1	s3312	PCSK7	Hs00237114_m1	S17509
Col6A2	Hs0154040_m1	s3314	*POSTN*	Hs01566734_m1	
Col6A3	Hs00914223_m1	s3317	*RUNX1*	Hs02558380_m1	s2469
CTGF	Hs00170014_m1		*SHROOM2*	Hs01113636_m1	
CREBBP	Hs00231733-m1	s3595	*SDC1*	Hs00896424-g1	
ELN	Hs00355783-m1		*SFRP4*	Hs00180066_m1	
ENPP1	Hs00355783-m1		*TEAD4*	Hs01125032_m1	s13964
EP300	Hs00914223_m1	s4695	*TNC*	Hs0115665_m1	
FAIM	Hs00202349_m1		*TGFB1*	Hs00998133_m1	
FN1	Hs01549976_m1		*VCAM1*	Hs01003372_m1	
FOSL1	Hs00759776_s1		*Non-targeting*		4390847
FOXM1	Hs01073586_m1				

SI Appendix, Table S5. Proteomic analysis of CBP30 treated cells

Accession	Gene Name	Peptide count	Unique peptides	Confidence score	Anova (p value)	Max fold change
Upregulated						
O95793;Q9NUL3	**STAU1**	4	1	298	3.23E-02	4.52
Q6ZVM7	**TOM1L2**	1	1	50	3.61E-03	3.90
Q9UK45	**LSM7**	3	3	114	3.85E-02	3.15
Q15397	**PUM3**	1	1	55	3.03E-02	2.66
Q9NUQ9;Q9H0Q0	**FAM49B**	2	2	118	3.88E-03	2.33
O43148	**RNMT**	1	1	45	2.17E-02	2.33
Q8TBF2	**FAM213B**	1	1	69	6.66E-03	2.24
P62140	**PPP1CB**	14	4	834	1.78E-02	2.03
O43567	**RNF13**	1	1	106	3.30E-02	1.88
Q8WW22	**DNAJA4**	2	1	80	2.94E-02	1.86
O60488	**ACSL4**	4	2	217	3.01E-02	1.86
Q0JRZ9	**FCHO2**	1	1	65	1.94E-03	1.85
Q14980	**NUMA1**	6	5	259	2.21E-02	1.75
P61018	**RAB4B**	2	1	91	4.18E-02	1.72
Q9Y6Y8	**SEC23IP**	8	7	367	4.52E-04	1.68
Q10713	**PMPCA**	2	2	96	2.97E-02	1.65
Q01105;P0DME0	**SET**	11	10	559	1.93E-02	1.64
Q9UBG0	**MRC2**	13	13	595	3.63E-03	1.61
O95479	**H6PD**	3	3	138	3.03E-02	1.61
Q9BWS9	**CHID1**	2	2	63	1.41E-02	1.57
P35222	**CTNNB1**	15	10	831	3.97E-02	1.55
O60716	**CTNND1**	9	9	487	1.12E-03	1.54
Q9BRF8	**CPPED1**	2	2	173	3.22E-04	1.54
Q7L311	**ARMCX2**	3	1	121	3.24E-03	1.53
A5YKK6	**CNOT1**	3	1	179	3.71E-02	1.51
Downregulated						
P42226	**STAT6**	2	1	75	1.58E-02	-1.50
P12109	**COL6A1**	29	28	1756	3.47E-03	-1.52
Q15582	**TGFBI**	3	3	126	4.84E-02	-1.59
Q9NQX4	**MYO5C**	1	1	33	3.81E-03	-1.62
Q7KZ85	**SUPT6H**	2	1	68	3.71E-02	-1.62
P12111	**COL6A3**	104	92	6341	1.58E-02	-1.62
O14495	**PLPP3**	2	2	78	3.64E-03	-1.66
P17096	**HMGA1**	1	1	72	3.74E-02	-1.70
P12110	**COL6A2**	20	18	1058	1.70E-03	-1.75
P02751	**FN1**	18	17	993	9.54E-03	-1.77
P28300	**LOX**	1	1	35	3.69E-05	-1.88
P32322	**PYCR1**	2	1	102	1.77E-02	-1.95
CG8	**CTHRC1**	1	1	23	2.00E-02	-2.53
P29373;P29762	**CRABP2**	6	6	266	4.16E-04	-2.95

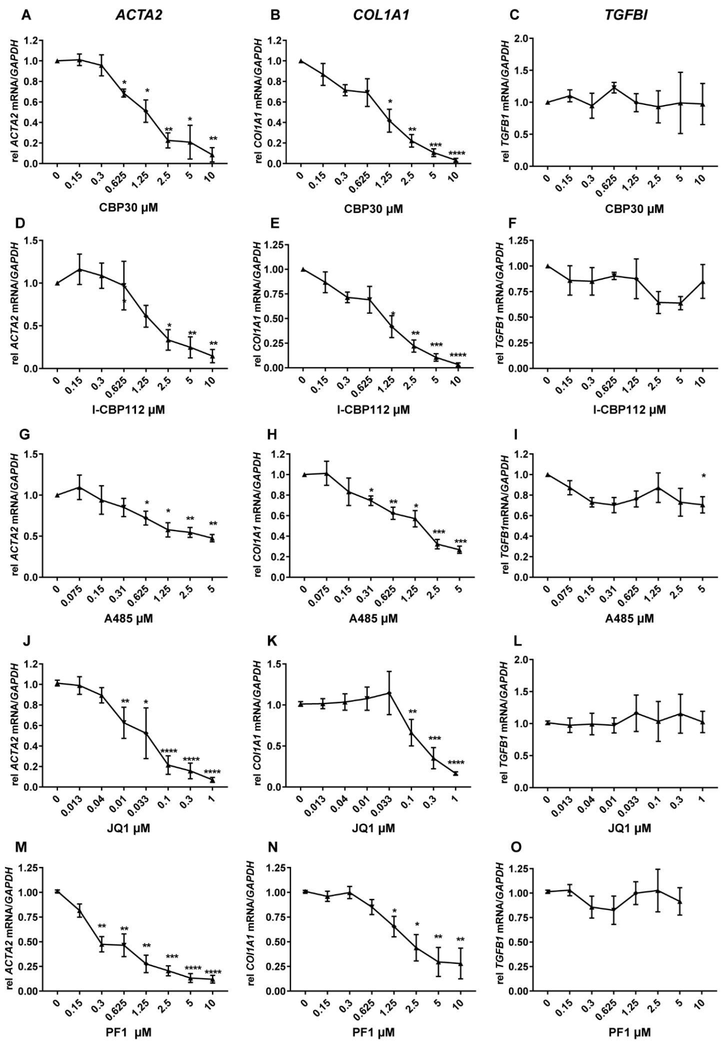

***SI Appendix* Figure S1.**

Dose dependent inhibition of *ACTA2* and *COL1A1* gene expression by bromodomain inhibitors**.** PCR analysis following 3 days exposure of myofibroblasts to either CBP30 (**A-C**), I-CBP112 (**D-F**), A485 (**G-H**), JQ1 (**J-L**) and PF1 (**M-O**). Gene expression was analysed by Taqman qPCR using the ΔΔ Ct method, normalised *GAPDH,* mean ± SEM of 4-6 donors. *P* value was determined by one sample *t* test to normalised DMSO of 1, * $P \leq 0.5$, ** $P \leq 0.01$, *** $P \leq 0.001$, **** $P \leq 0.0001$.

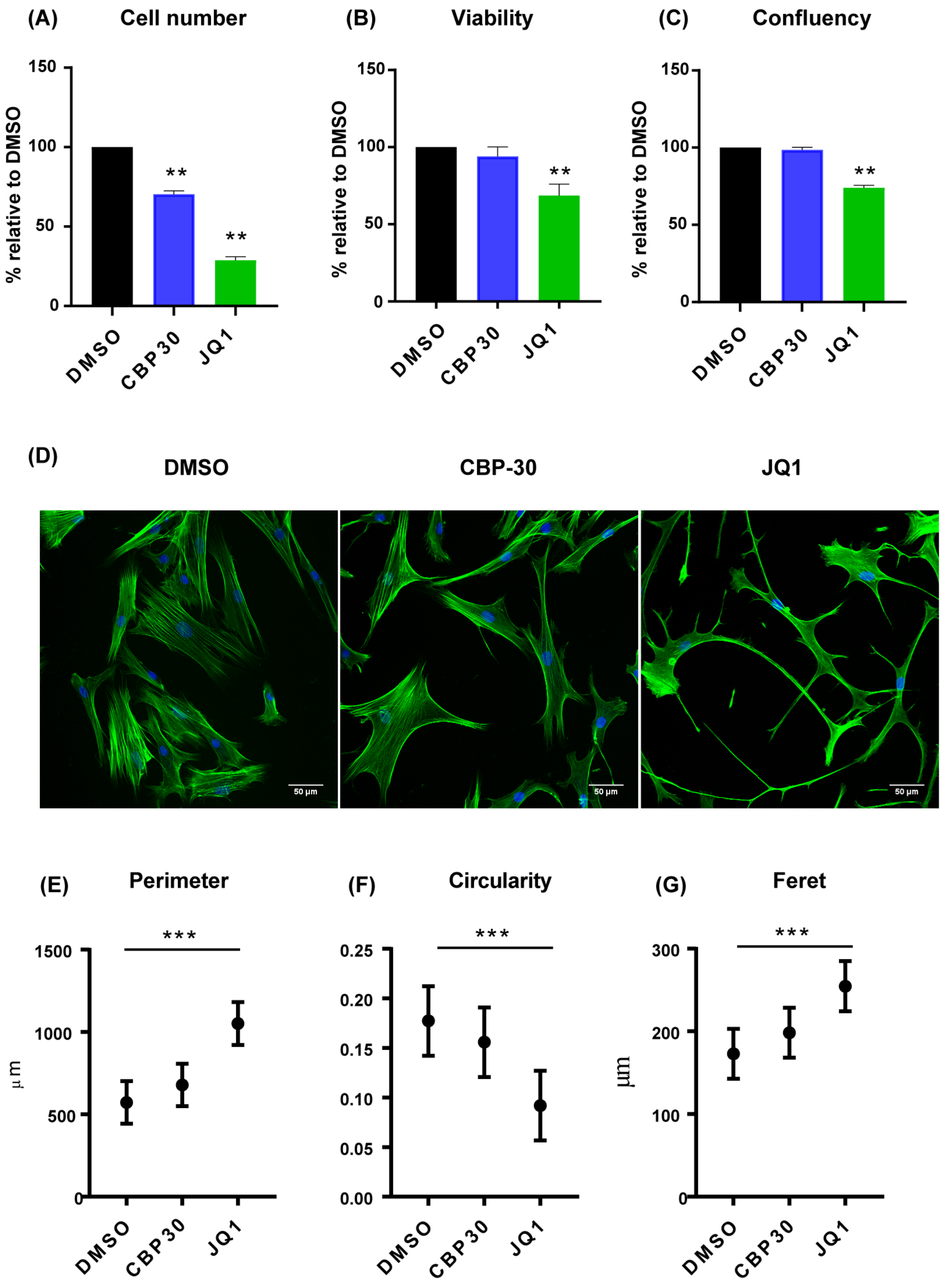

(A)
Cell number
% relative to DMSO
150
100
50
0
**
**
DMSO
CBP30
JQ1
(B)
Viability
% relative to DMSO
150
100
50
0
**
DMSO
CBP30
JQ1
(C)
Confluency
% relative to DMSO
150
100
50
0
**
DMSO
CBP30
JQ1
(D)
DMSO
CBP-30
JQ1
50 µm
50 µm
50 µm
(E)
Perimeter
***
µm
1500
1000
500
0
DMSO
CBP30
JQ1
(F)
Circularity
***
0.25
0.20
0.15
0.10
0.05
0.00
DMSO
CBP30
JQ1
(G)
Feret
***
µm
300
200
100
0
DMSO
CBP30
JQ1

***SI Appendix* Figure S2.**

Pharmacological targeting of CBP/EP300 alters myofibroblast morphology. **(A)** Myofibroblast were treated with DMSO, JQ1 (0.5µM) and SGC-CBP30 (2.5µM) for 3 days. The effects of the drugs on cell morphology was determined following phalloidin staining of actin filaments was quantified (**B-D**) from 5 donors using a minimum 50 cells per donor with ImageJ and analysed with a Mixed Linear Model with Tukey Post-hoc Test *** $P = <0.001$ (*Circularity:* $4\pi \times ([Area] / [Perimeter]^2)$ with a value of 1.0 indicating a perfect circle.). Viability assays were performed on 4 donors using of Hoechst and Calcein stained cells, quantified using the Celigo Image cytometer. Mean ± SEM of 4 donors *P* value was determined by one sample *t* test to normalised DMSO of 1. * $P \leq 0.5$, ** $P \leq 0.01$, *** $P \leq 0.001$.

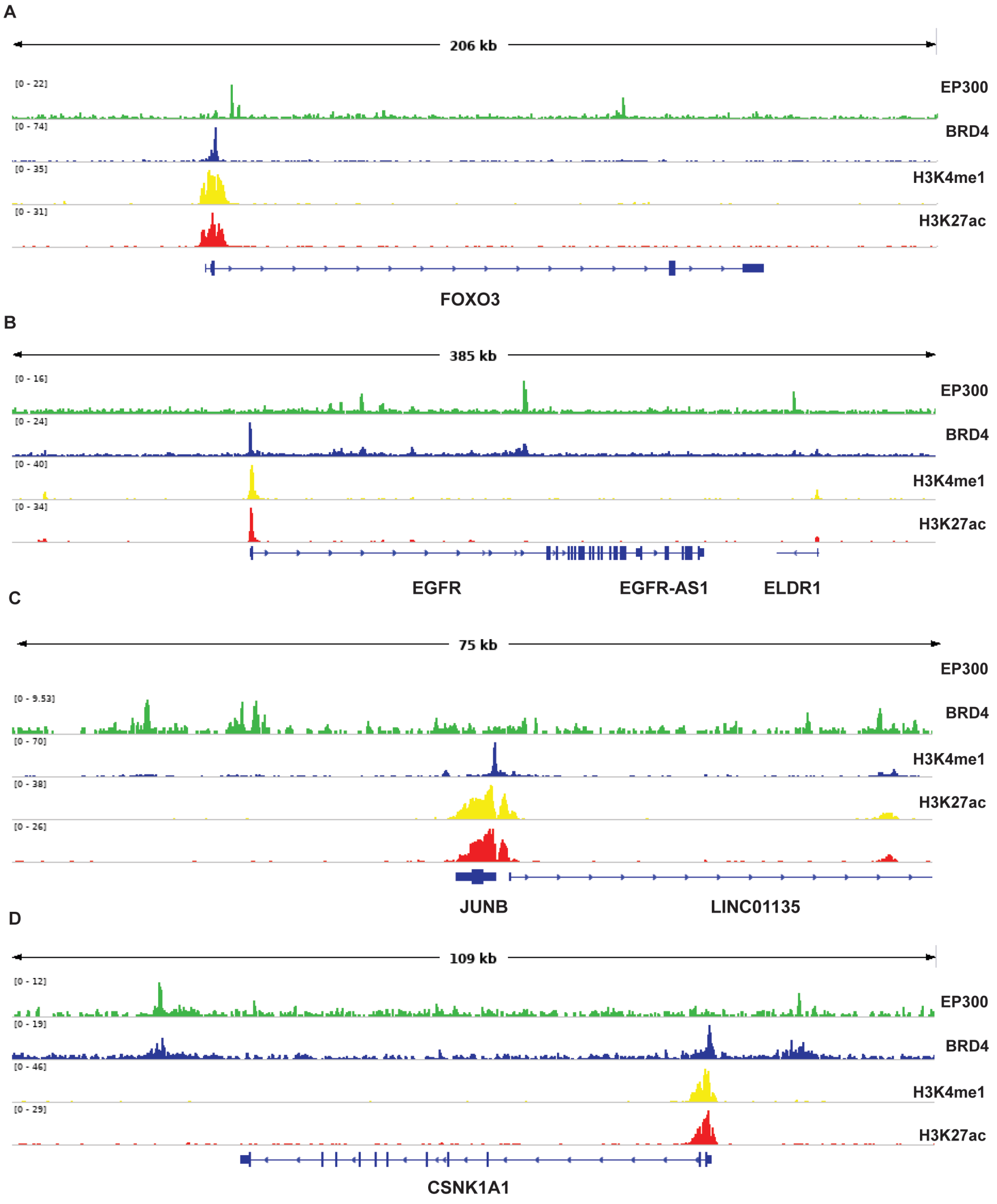

***SI Appendix,* Figure S3.**

Binding of EP300 at genomic locations of FoxO and WNT pathway genes.

Visualisation in IGV of ChIP-Seq signal at the loci of *FOXO3, EGFR, JUNB* and *CSK1A1*, autoscaling was used, and all files normalised to input.

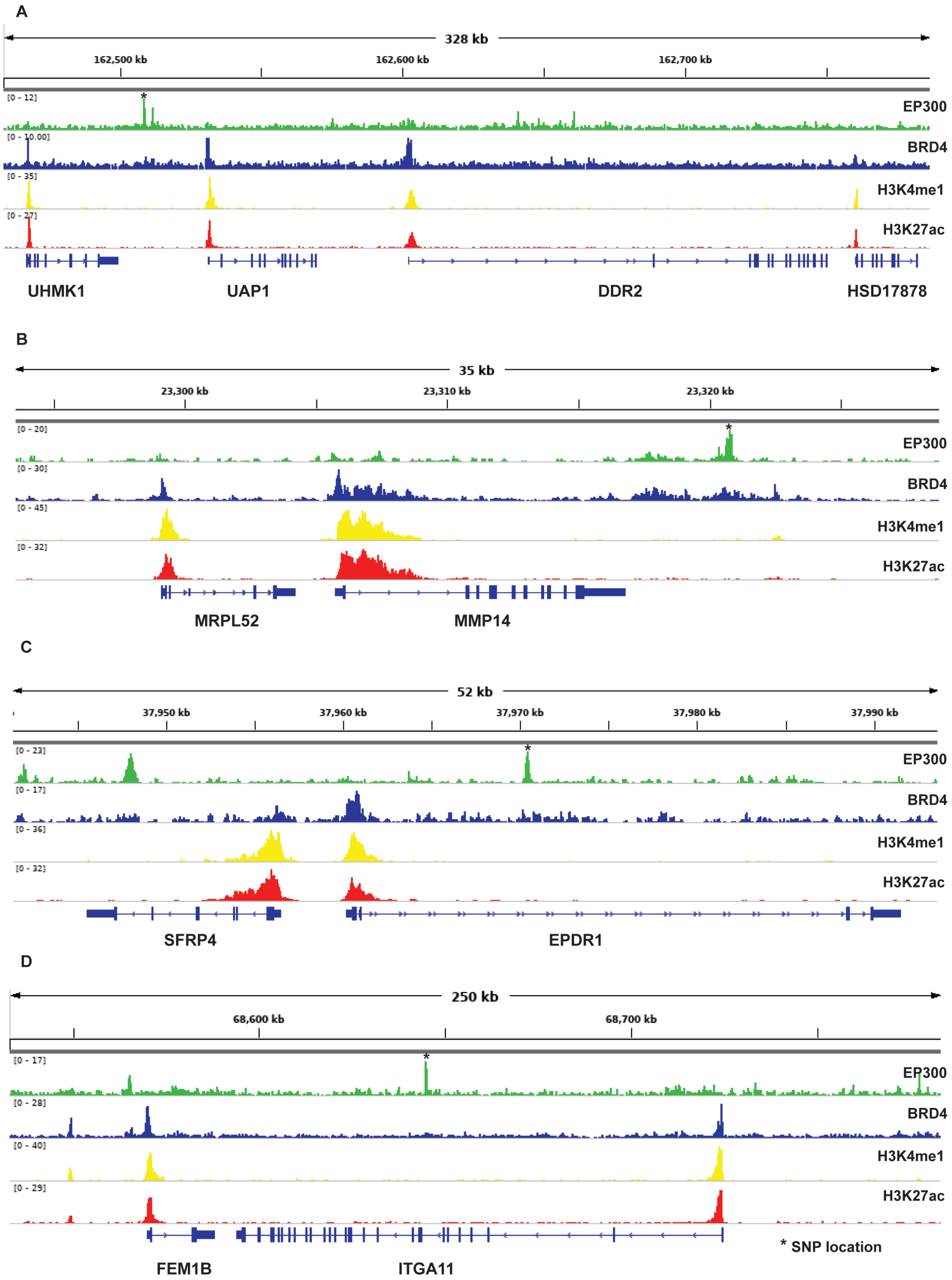

***SI Appendix*, Figure S4. Binding of EP300 at genomic locations of SNPs significantly associated with Dupuytren's Disease.** Visualisation in IGV of EP300 ChIP-Seq signal at the loci of *DDR2, MMP14, SFRP4* and *ITGA11*, autoscaling was used and all files normalised to input.

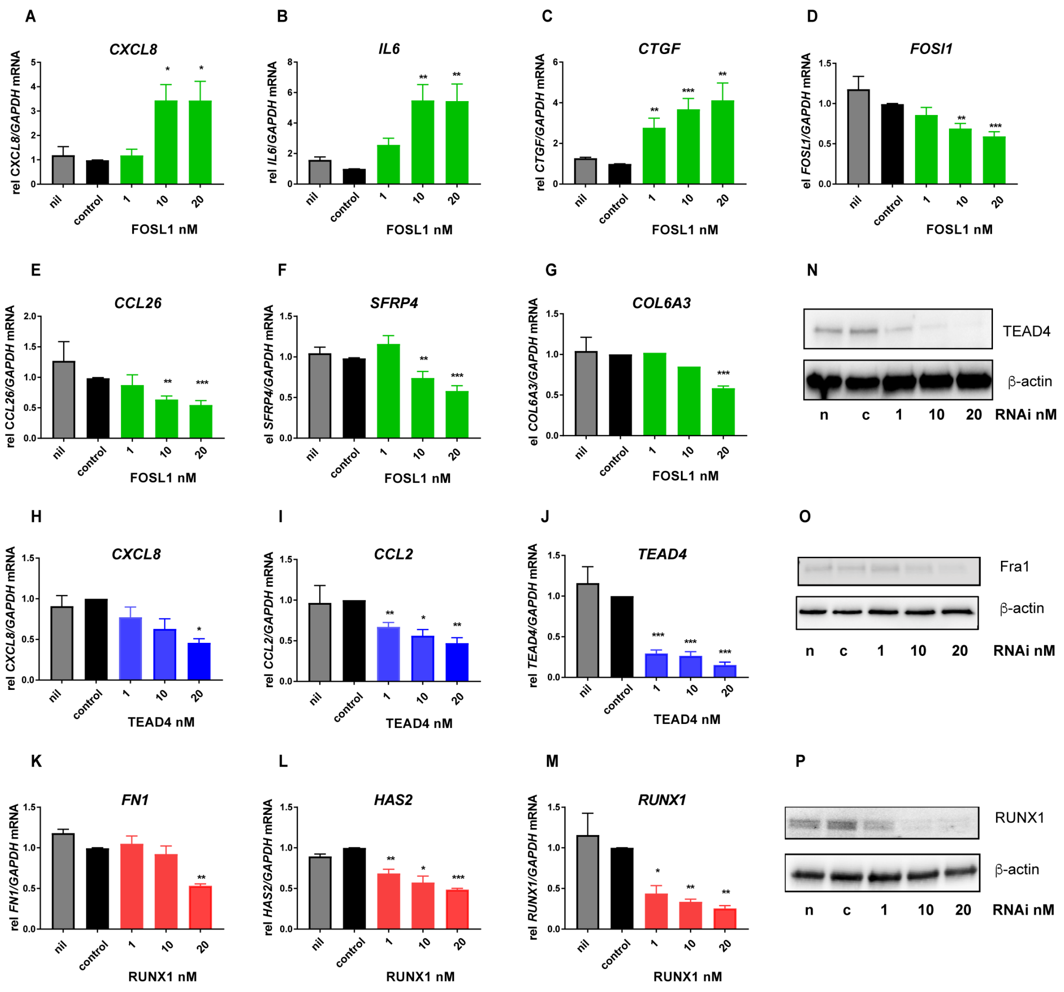

SI Appendix, **Figure S5.**

FOSL1, TEAD4 **and** ***RUNX1*** **depletion highlight differential regulation of specific subsets of genes.** PCR analysis of 3 day siRNA (20nM) mediated depletion studies show Fra1 regulates *A CXCL8* (**A**), *IL-6 (***B**), *CTFG* (**C**), *FOSL1 (D), CCL26 (E), SFRPF4 (F)* and *COL6A3* (**G**)**.** Gene expression was analysed by Taqman qPCR using the ΔΔ Ct method, normalised *GAPDH.* TEAD4 depletion regulates the gene expression of the chemokines *CXCL8 (H)* and *CCL2 (I)* and *TEAD*4. (**J**). *RUNX1* depletion regulates the gene expression of ECM components *FN1* (**K**) and *HAS2* (**L**) and *RUNX1* (**M**). Mean ± SEM of 4 donors *P* value was determined by one sample *t* test to normalised control non-targeting oligo value of 1. * $P \leq 0.5$, ** $P \leq 0.01$, *** $P \leq 0.001$. Western

blot analysis confirms knockdown at the protein level of FOSL1/Fra1 (**N**), TEAD4 **(O)** RUNX1 (**P**) representative of 3 experiments**.**

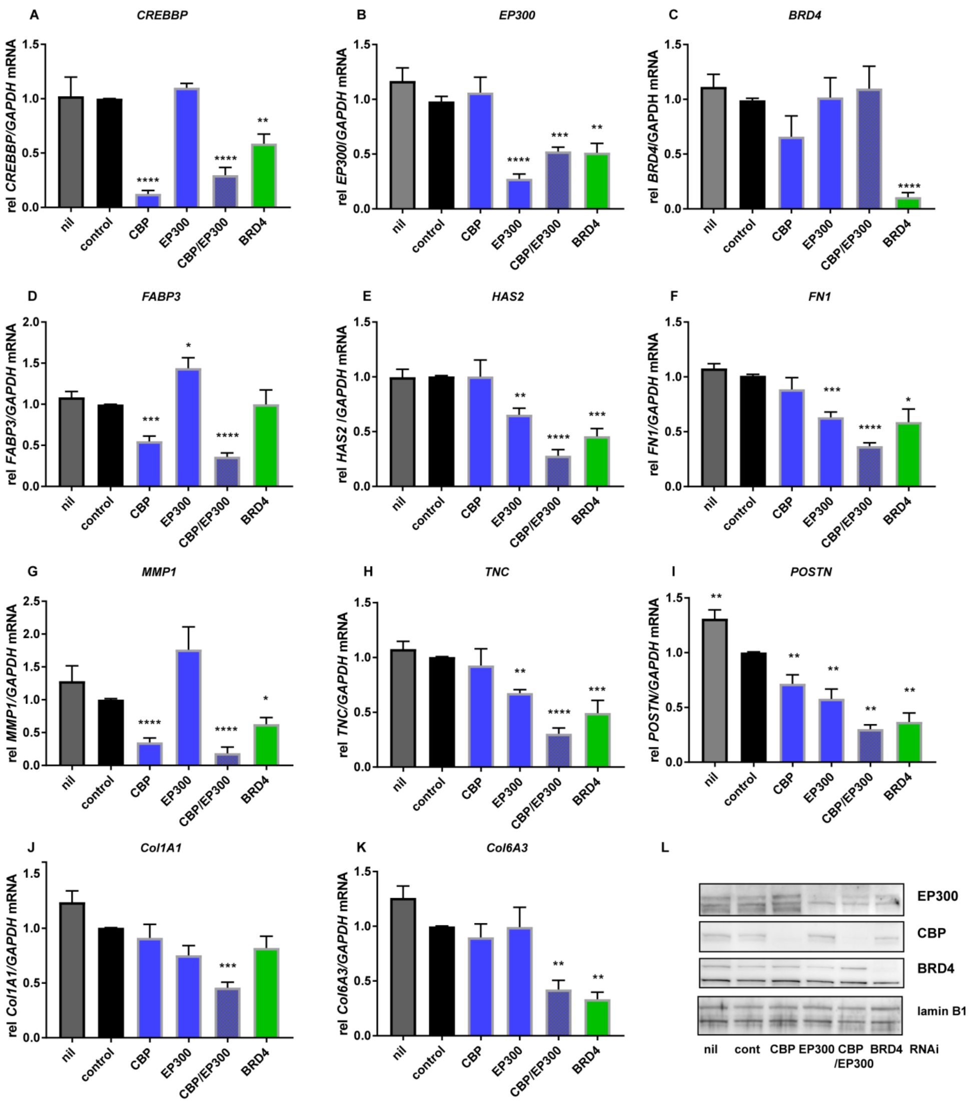

SI Appendix, **Figure S6.**

CREBBP/CBP, EP300 and BRD4 siRNA mediated validation of RNA-Seq data**.** PCR analysis following 4 day depletion of either CBP (20nM), EP300 (20nM) or a combination of CBP/EP300 (10nM each), BRD4 or a non-targeting control oligo siRNA (20nM) (**A**) *CREBBP,* (**B**) *EP300*, (**C***) BRD4*, and confirms regulation of *FABP3* (**D**), *HAS2* (**E**), *FN1* (**F**), *MMP1* (**G**), *TNC* (**H**),

POSTN (**I**), *COL1A1* (**J**) and *COL6A3*(**K**). Gene expression was analysed by Taqman qPCR using the ΔΔ Ct method, normalised *GAPDH,* mean ± SEM of 6 donors, *P* value was determined by one sample t test to normalised control non-targeting oligo value of 1. * $P \leq 0.05$, ** $P \leq 0.01$, *** $P \leq 0.001$. Western blot analysis confirms knockdown at the protein level of EP300, CBP and BRD4 (**L**) representative of 3 donors.

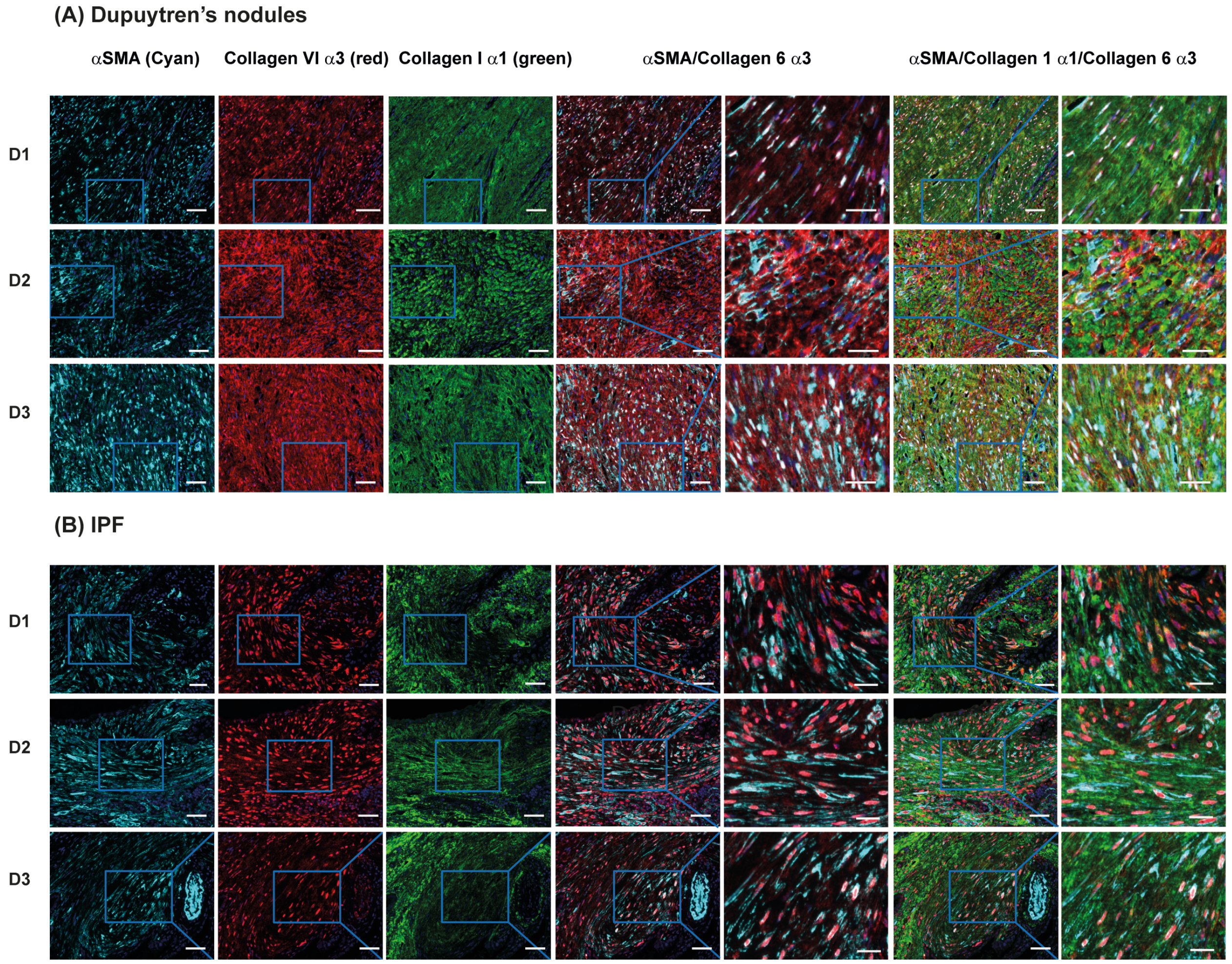

SI Appendix, **Figure S7.**

Immunofluorescence staining of 3 further DD and IPF patient samples. Confocal microscopy after immunostaining using anti-αSMA, anti-Collagen VI α3 or anti-Collagen 1 α 1 specific antibodies in either (A) DD nodule or IPF (B) sections, shown are 3 (D1-3) donors, scale bar 25 or 50 μM for higher magnification slides.

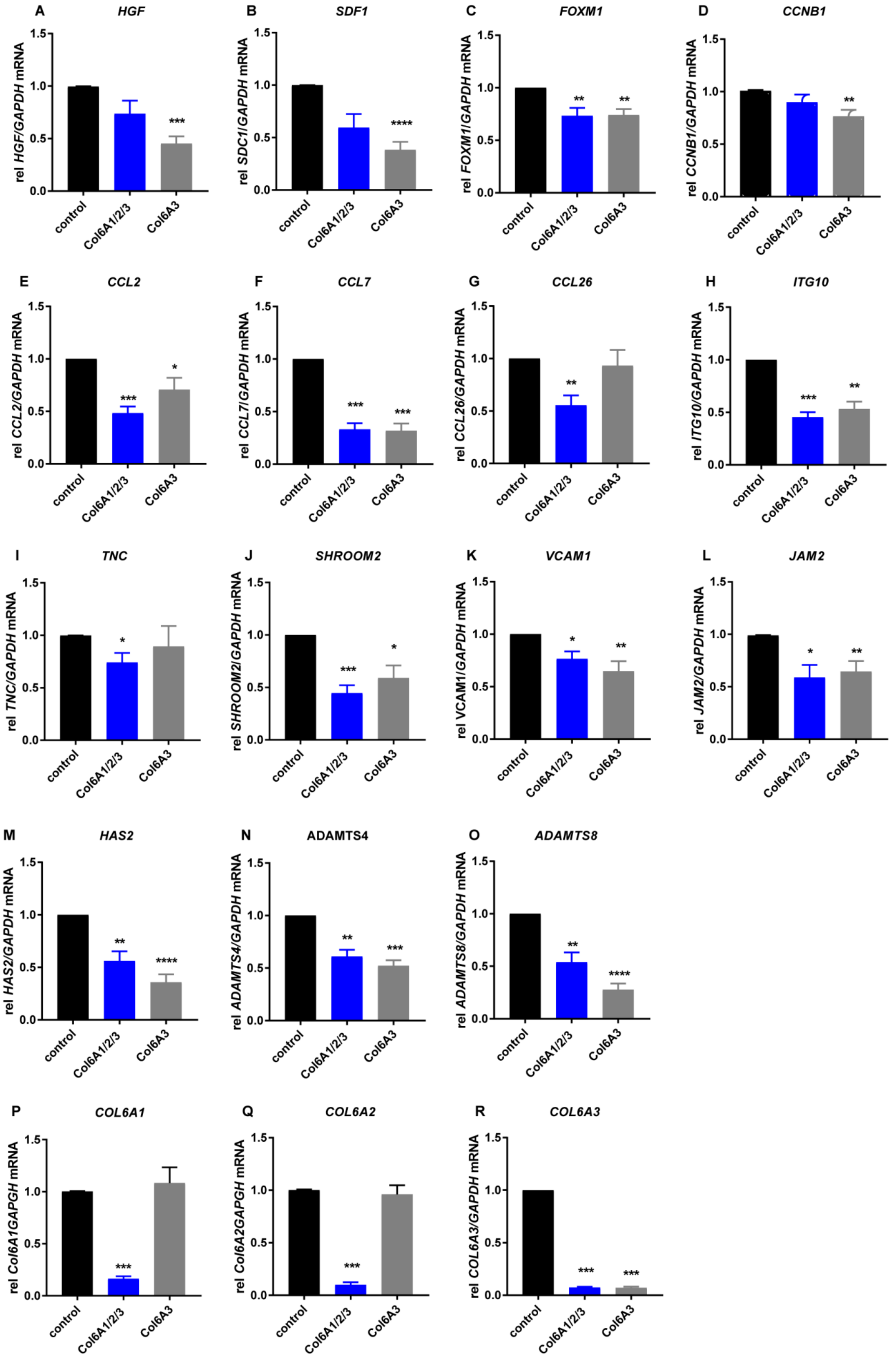

SI Appendix, **Figure S8.**

PCR validation of *COL6A1/2/3* siRNA mediated validation of RNA-Seq data. PCR analysis following 6 day depletion of COL6A1/2/3, COL6A3 or a non-targeting control oligo siRNA

(20nM) effects on (**A**) *HGF* (**B**) *SDC1* (**C**) *FOXM1*(**D**), *CCNB1*(**E**), *CCL2,* (**F**) CCL7, (**G**) CCL26, (**H**) *ITG10,* (**I**) *TNC,* (**J**) *SHROOM2*, (**K**) *VCAM1*, (**L**) *JAM2*, (**M**) *HAS2*, (**N**) *ADAMTS4*, (**O**) *ADAMTS8*, (**P**) *COL6A1*, (**Q**) *COL6A2* and (**R**) *COL6A3* . Gene expression was analysed by Taqman qPCR using the ΔΔ Ct method, normalised *GAPDH*. Mean ± SEM of 6 donors, P value was determined by one sample *t* test to normalised control non-targeting oligo value of 1. * $P \leq 0.05$, ** $P \leq 0.01$, *** $P \leq 0.001$.

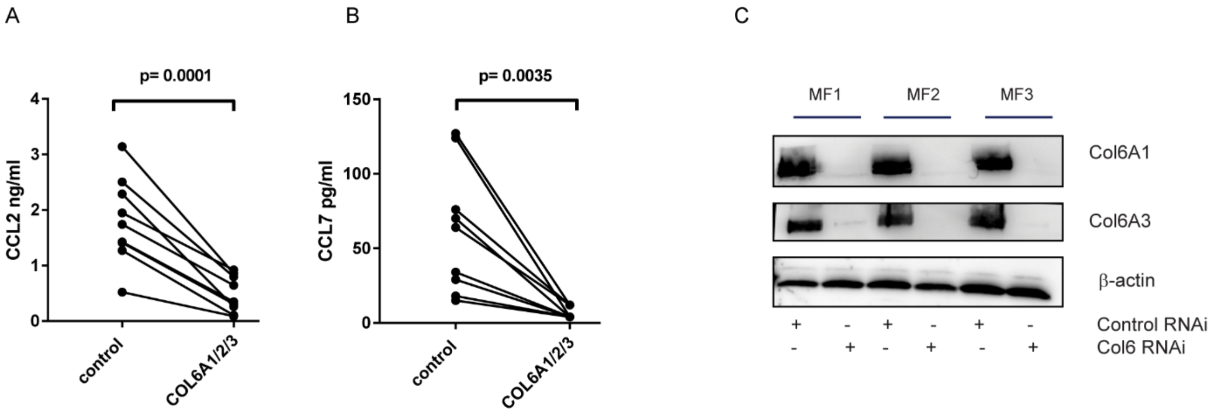

***SI Appendix* Figure S9.**

COL6A1/2/3 siRNA depletion inhibits CCL2 and CCL7 secretion in DD myofibroblasts. Cells were treated with either *COL6A1/2/3* or a non-targeting control oligo siRNA (20nM) for 6 days and effects on CCL2 and CCL7 secretion were determined by ELISA. P value was determined by paired sample *t* test in 8 donors. (**B**) Western blot analysis confirms knockdown at the protein level of Col6A1 and Col6A3 in 3 donors, β- actin confirms equal loading.

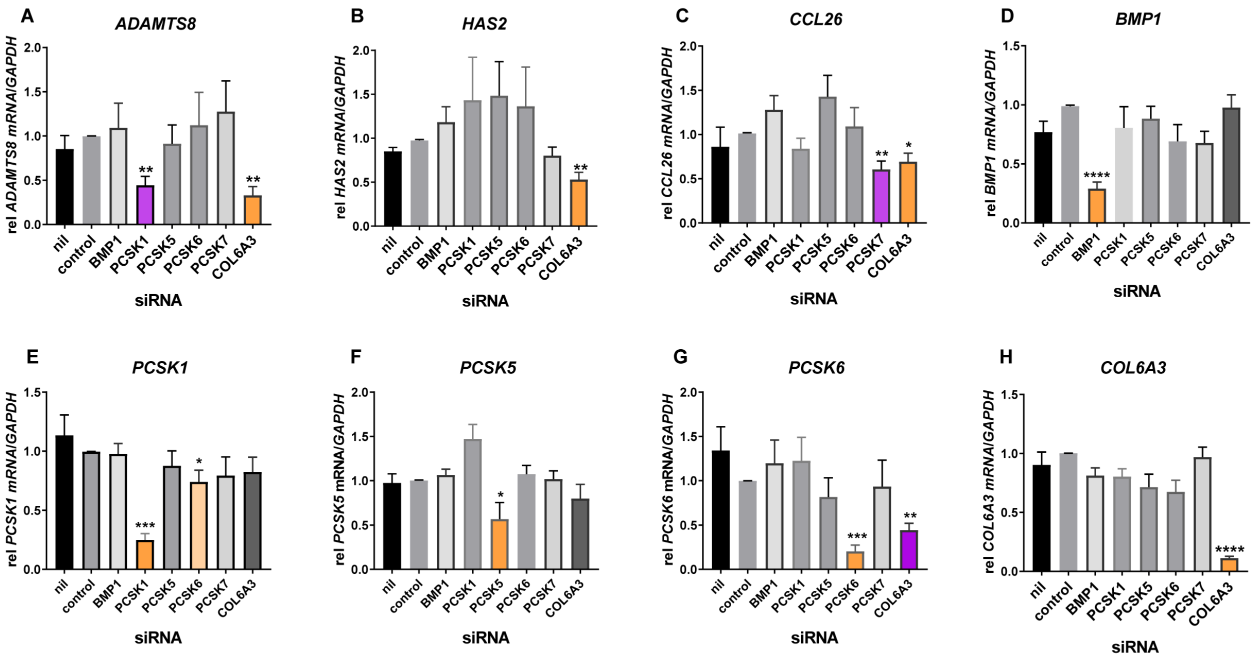

***SI Appendix,* Figure S10.**

PCR validation of *PCSK* depletion**.** PCR analysis following 6 day depletion of PCSKs or a non-targeting control oligo siRNA (20nM) effects on (**A**) *ADAMTS8*, (**B**) *HAS2* (**C***) CCL26,* (**D**) *BMP1*, (**E**) *PCSK1,* (**F**) *PCSK5*, (**G***) PCSK6* and (**H**) *COL6A.* Gene expression was analysed by Taqman qPCR using the ΔΔ Ct method, normalised to *GAPDH*. Mean ± SEM of 6 donors, *P* value was determined by one sample *t* test to normalised control non-targeting oligo value of 1. * $P \leq 0.05$, ** $P \leq 0.01$, *** $P \leq 0.001$, **** $P \leq 0.0001$.
